# Discovery of benzothiazolylquinoline conjugates as novel human A_3_ receptor antagonists: biological evaluations and molecular docking studies

**DOI:** 10.1098/rsos.171622

**Published:** 2018-02-07

**Authors:** Bidisha Sarkar, Santanu Maiti, Gajanan Raosaheb Jadhav, Priyankar Paira

**Affiliations:** Department of Chemistry, School of Advanced Sciences, VIT University, Vellore, Tamil Nadu 632014, India

**Keywords:** benzothiazolylquinoline, human A3 receptor antagonist, molecular docking, radioligand binding assay, glide score

## Abstract

Adenosine is known as an endogenous purine nucleoside and it modulates a wide variety of physiological responses by interacting with adenosine receptors. Among the four adenosine receptor subtypes, the A_3_ receptor is of major interest in this study as it is overexpressed in some cancer cell lines. Herein, we have highlighted the strategy of designing the *h*A_3_ receptor targeted novel benzothiazolylquinoline scaffolds. The radioligand binding data of the reported compounds are rationalized with the molecular docking results. Compound **6a** showed best potency and selectivity at *h*A_3_ among other adenosine receptors.

## 1. Introduction

Adenosine is an endogenous purine nucleoside which regulates many physiological functions through the activation of four specific receptor subtypes, classified as A_1_, A_2A_, A_2B_ and A_3_ adenosine receptors (AR), belonging to the family of G-protein-coupled receptors [1]. These receptors are widely distributed in mammalian tissues. The A_3_AR subtype is the most recently characterized member of the family which was first cloned from a rat testis cDNA library [2] and is still undergoing intensive pharmacological characterization. The A_3_AR subtype is implicated in various pathological conditions such as cardiac and cerebral ischaemia, neurodegenerative diseases as well as inflammatory pathologies including rheumatoid arthritis and asthma [3]. Furthermore, A_3_AR is overexpressed in various neoplastic cells including HL-60 leukaemia, human Jurkat T-cell lymphoma, astrocytoma, A378 melanoma, B16-F10 and solid tumour (e.g. a two–threefold increase in colon carcinomas), while low or almost no receptor expression was found in normal cells [4,5].

Similar results were found in studies of the receptor expression levels in tumour tissues derived from patients with colon, breast, small cell lung, pancreatic and hepatocellular carcinomas and melanoma in direct comparison with adjacent body normal tissues [6–15]. Higher A_3_AR expression in the tumour versus adjacent non-neoplastic tissue was further confirmed by reverse transcription-PCR analysis of colon and breast carcinoma. Protein analysis of A_3_AR expression in fresh tumours derived from colon (*n *= 40) or breast (*n = *17) revealed 61% and 78% higher expression in the tumour than adjacent normal tissue, respectively [16]. Thus the A_3_ receptor could be a prospective therapeutic target and biological predictive marker in cancer therapy. The high A_3_AR expression level in the tumour tissues was associated with elevated nuclear factor *κ*B and cyclin D1 levels [16]. High A_3_AR mRNA expression was also exhibited in other solid tumour types. Mechanistic studies demonstrated that A_3_AR activation by synthetic agonists or antagonists induces down-regulation of key cell growth-regulatory proteins including cyclin D1 and nuclear factor *κ*B [10,17,18]. Hence, discovery of selective A_3_AR targeting ligands has been a great challenge in last two decades. Moreover, A_3_AR antagonists research not only aids in the development of therapeutic agents but also in the development of diagnostic agents [2,19]. Nowadays, the diagnostic approaches have been significantly developed by using fluorescently labelled pharmacophores.

In the past few years, there have been strenuous efforts to develop different heterocyclic scaffolds as *h*A_3_antagonists including pyridine and dihydropyridine analogues, flavonoid, isoquinoline, triazoloquinazolines, pyrazolo-[3,4-*c*]or-[4,3-*c*]quinolones, triazoloquinoxaline, pyrazolo-[4,3-*e*]1,2,4-triazolo-[1,5-c]pyrimidines, ruthenium-pyrazolopyrimidines [20–29]. However, none of the pharmacophores has been tested in A_3_ overexpressed cancer cell lines. The cellular imaging study and cell surface receptor localization study was also not performed because these scaffolds are nonfluorescent. Therefore, to identify the *h*A_3_ targeting novel fluorescent ligands, a molecular simplification followed by molecular docking approach was employed.

**Figure 1.**
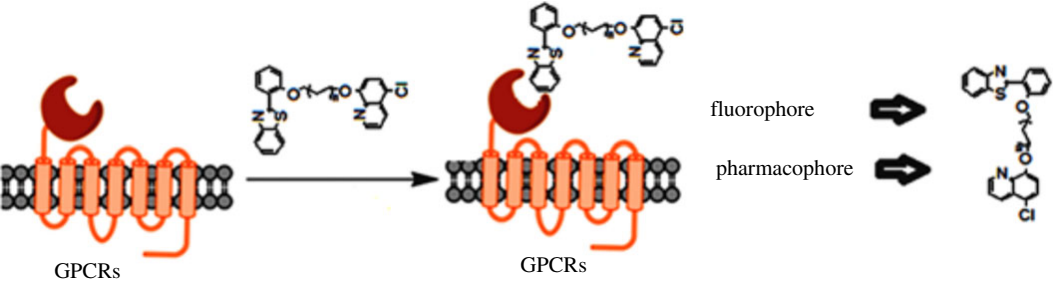
Dual role: pharmacophore as well as a fluorophore. G-PCR, G-protein coupled receptor.

Benzothiazole possesses several biological activities such as anti-inflammatory, antimicrobial, anti-HIV, anticancer and amyloid marker [30–36]. These scaffolds are also able to arrest metal promoted amyloid fibril build-up [37]. Likewise, 8-hydroxy quinoline has also been developed as potent bioactive scaffold [38]. Herein, we have highlighted the strategy of designing the novel 2-(2′-hydroxyphenyl)benzothiazole (HBT) scaffolds having dual properties (pharmacophore and fluorophore) via molecular simplification followed by a molecular docking approach (figure 1). The designed molecules have already shown potency and selectivity in *h*A_3_AR overexpressed cancer cell lines than normal cell lines [38]. In our earlier report, the cellular localization was also observed using those scaffolds. To justify the molecular pathway of these drugs, we have initiated the molecular docking approach as well as radioligand binding study at *h*A_3_AR.

## 2. Results and discussion

### 2.1. Chemistry

#### 2.1.1. Molecular simplification

**Figure 2.**
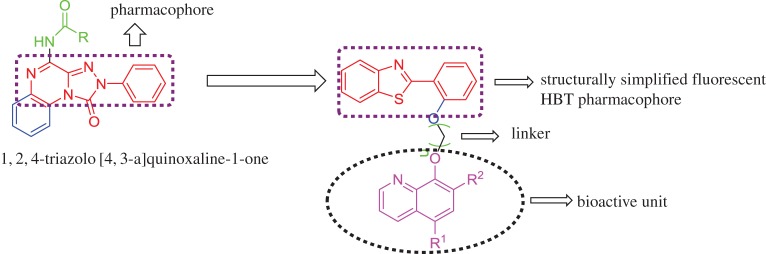
Molecular simplification approach to design the *h*A_3_ targeting ligand.

Following a molecular simplification approach, we have identified the benzothiazolylquinoline ring system as an appropriate core skeleton for the design of novel fluorescent *h*A_3_AR antagonists. Here, we have simplified the structure of the tricyclic A_3_ antagonist (triazoloquinoxaline) into bicyclic motif (2-benzothiazolyl phenol). In the course of structure design, we have attached the bicyclic fluorescent pharmacophore (2-benzothiazolyl phenol) with another bioactive 8-hydroxy quinolone unit through a linker to enhance the A_3_ binding ability (figure 2).

### 2.2. Synthesis

**Scheme 1.**
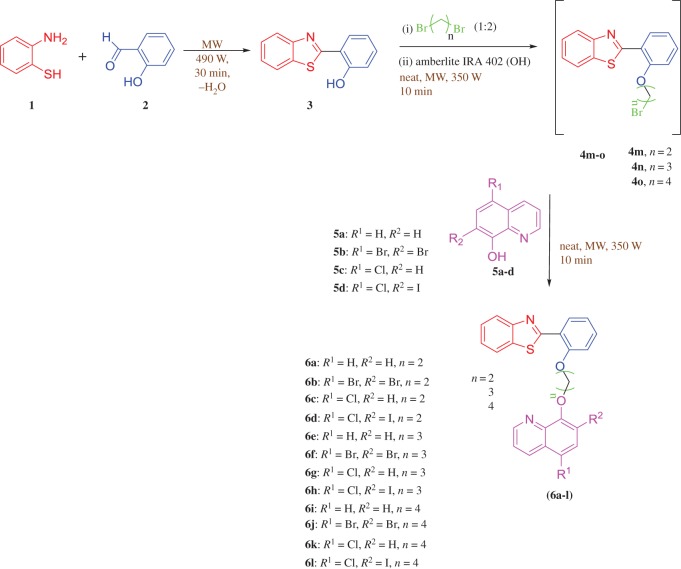
Preparation of 8-[2-(2-benzothiazol-2-yl-phenoxy)-alkoxy]-quinoline derivatives (**6a-l**) [38].

**Figure 3.**
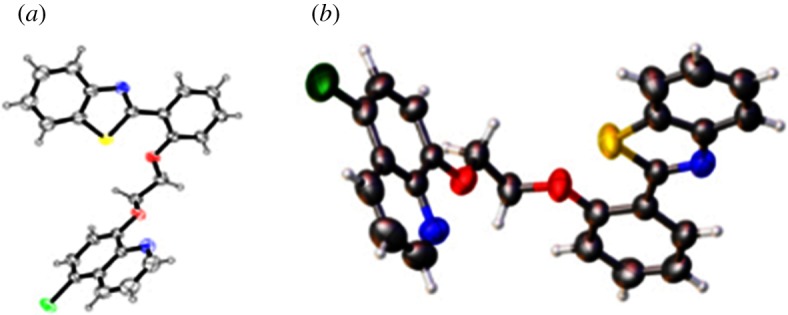
ORTEP diagrams of compound **6c** drawn at the 50% probability level [38].

In our earlier report, a series of 2-(2′-hydroxyphenyl)benzothiazolylquinoline scaffolds were prepared in conventional way and also under microwave condition in one pot sequence (scheme 1) [38]. We followed the earlier reported green method to synthesize the following scaffolds and then characterized by ^1^H NMR, ^13^C NMR and LCMS study (electronic supplementary material, supporting information). Structure of compound **6c** was further confirmed by single crystal X-ray study (figure 3) [38].

### 2.3. Molecular docking studies

#### 2.3.1. Homology modelling

The crystallographic structure of *h*A_2A_AR complexed with ZM-241385 as a high affinity antagonist (PDB code: 3EML) [39]. It was already being used to build up a homology model of the *h*A_3_AR by our group [40]. Considering the high resolution (2.6 Å) and accuracy of the structure of *h*A_2A_AR, 3EML was selected as a template by various research groups [41,42]. Modeller 9.11 was used to perform the homology modelling [43–46] and the quality of the model was evaluated using the Ramachandran plot. Subsequently, the prediction ability of the constructed *h*A_3_AR homology model was evaluated in the molecular docking experiments using the GLIDE tool from Schrödinger maestro.

#### 2.3.2. Molecular docking

Our approach was to select a new class of *h*A_3_AR targeting compounds from a group of hypothetically designed 2-phenylbenzothiazole (HBT)-based scaffolds. We performed molecular docking of 12 different HBT-based ligands using the GLIDE tool from Schrödinger maestro to identify the hypothetical binding mode at the *h*A_3_AR. Finally, we have identified the best inhibitor for targeted *h*A_3_AR from GLIDE scores (table 1).

**Table 1.** Binding energy of benzothiazolylquinoline analogues (**6a-l**) with *h*A_3_.

entry	compound	GLIDE score (Kcal mol^−1^)
1	**6a**	−10.31
2	**6b**	−9.06
3	**6c**	−6.50
4	**6d**	−8.90
5	**6e**	−9.05
6	**6f**	−8.81
7	**6g**	−9.04
8	**6h**	−8.78
9	**6i**	−7.48
10	**6j**	−8.37
11	**6k**	−9.05
12	**6l**	−9.36

**Figure 4.**
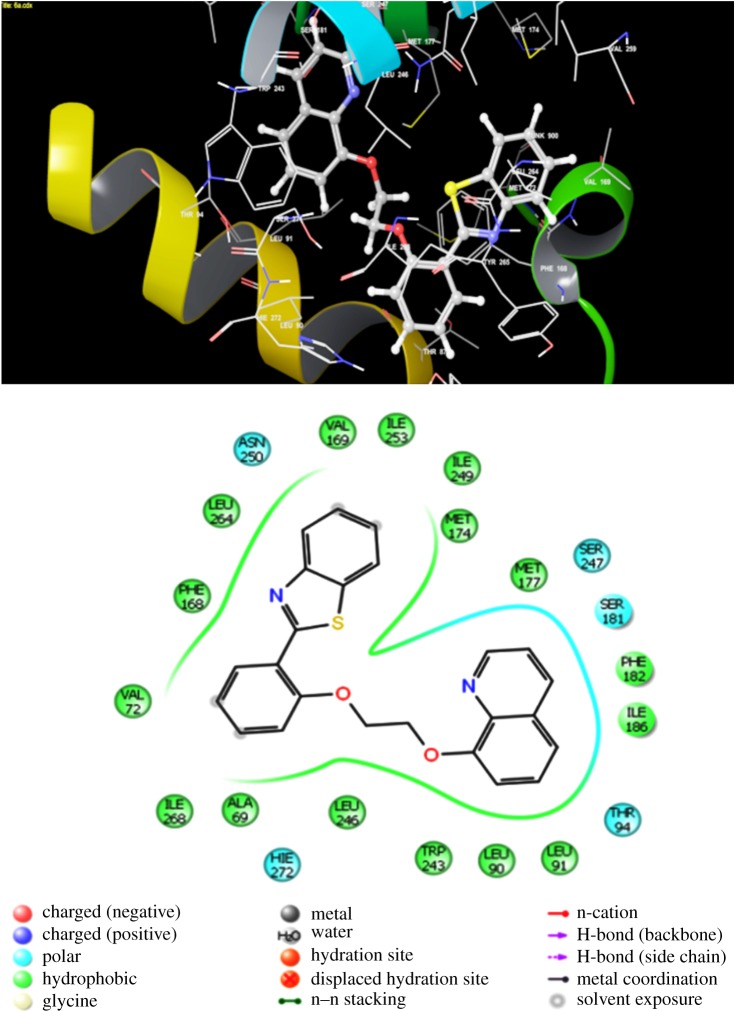
Binding interaction of most potent benzothiazolylquinoline analogue (**6a**) with *h*A_3_ receptor.

All the newly synthesized benzothiazolylquinoline scaffolds were docked into the orthosteric transmembrane-binding cavities of *h*A_3_AR. From the ligand docking, we have inferred that out of 12 synthesized scaffolds, compound **6a** displayed the best GLIDE score with the lowest binding energy. In figure 4, the hypothetical binding pose of compound **6a** is clearly observed at the *h*A_3_AR. In particular, the most prominent aromatic π–π stacking interactions are established between Phe 168 and ligands and it was anchored properly within the binding cleft. Moreover, a strong hydrogen bond with Phe 168 was also appeared within the binding pocket. Likewise, a strong π–π stacking interaction was observed between Tyr 265 and compound **6f**. Compound **6g** also formed a hydrogen bond with Val 169 (electronic supplementary material, figure S1)

#### 2.3.3. Binding affinity at *h*A_1_AR, *h*A_2A_AR, *h*A_2B_AR and *h*A_3_AR

The receptor binding affinities of the synthesized benzothiazolylquinoline derivatives (**6a-l**) are recapitulated in table 2. The binding affinity of the antagonist was estimated by measuring the displacement of selective radioligands which were formerly bound to the receptor expressed (Chinese hamster ovary cells (CHO) for *h*A_1_AR, *h*A_2A_AR and *h*A_3_AR) at the surface of the cell. In this particular assay, the displacement of: (i) specific [3H] CCPA (2-chloro-*N*(6)-cyclopentyladenosine) binding at *h*A_1_AR, (ii) specific [3H] NECA (5'-*N*-ethylcarboxamidoadenosine) binding at the *h*A_2A_AR, and (iii) [3H] HEMADO (2-(1-hexynyl)-*N*^6^-methyladenosine) at the *h*A_3_AR were evaluated. There is no suitable radioligand for *h*A_2B_AR found and hence the antagonists activity was determined in adenylyl cyclase experiments in CHO cells expressing the *h*A_2B_AR [47,48]. *K*_i_ (dissociation constant) value of the data was calculated using the Cheng and Prusoff equation [49], with geometric means of at least three experiments including 95% confidence intervals. From table 2, it was observed that most of the compounds exhibited a *K*_i_ value at *h*A_3_ in the range of 2–6 µM. Compound **6c** and **6i** showed least binding efficacy with *h*A_3_ which is clearly rationalized with their binding energy profile (GLIDE score). Compound **6a** exhibited the most binding potency at *h*A_3_ in the 2.6 µM range. This compound has also shown 38-fold and 11-fold more selective potency at *h*A_3_ than *h*A_1_ and *h*A_2A_, respectively. While increasing the length of the –CH_2_– linker from 2 to 3, binding potency of the compound **6e** at *h*A_3_ is reduced to 3.8 µM. Selectivity of this compound at *h*A_3_ with respect to *h*A1 and *h*A_2_ has also been reduced to some extent. Consequently, compound **6i** showed much less potency and selectivity in *h*A_3_. Compounds (**6f-h** and **6j-l**) having electronegative groups and lengthy linkers (*n* = 3, 4) showed good potency and selectivity at *h*A_3_. It was also observed that compound **6b** having two electronegative bromine groups with a small linker (*n* = 2) exhibited good potency (3.2 µM) and selectivity at *h*A_3_.

**Table 2.** Binding affinity (*K*_i_) of synthesized compounds at *h*A_1_AR, *h*A_2A_AR and *h*A_3_AR and selectivity against *h*A_1_AR and *h*A_2A_AR.

	*K*_i_, µM (95% CI)^d^				selectivity	
compound	*h*A_1_^b^	*h*A_2A_^c^	*h*A_2B_^a^	*h*A_3_^e^	*h*A_1_/A_3_*h*A_2A_/A_3_	
**6a**	>100	23.8 (21.6–26.4)	>30	2.6 (1.8–4.5)	>38.46	>11.53
**6b**	>100	17.2 (15.3–19.4)	>20	3.2 (2.4–4.1)	>31.25	>6.25
**6c**	30.8 (26.5–32.7)	32.8 (28.7–34.1)	>20	30.4 (27.6–32.1)	1.01	>19.80
**6d**	>100	>20	>20	5.6 (3.8–7.1)	>17.85	>3.57
**6e**	>100	>20	>30	3.8 (2.6–4.7)	>26.31	>7.89
**6f**	>100	>20	>30	6.2 (4.8–7.1)	>16.12	>4.83
**6g**	>100	>20	>20	4.2 (3.1–5.4)	>23.80	>4.76
**6h**	>100	>20	>20	6.1 (4.9–7.2)	>16.39	>3.27
**6i**	29.7 (27.8–32.6)	34.9 (32.7–37.1)	>20	25.4 (23.6–27.1)	1.16	>0.78
**6j**	>100	>20	>20	6.4 (4.9–8.2)	>15.62	>3.15
**6k**	>100	>20	>10	3.7 (2.7–4.8)	>27.02	>2.70
**6l**	>100	>20	>30	3.6 (2.1–4.8)	>27.77	>8.33

^a^Adenylyl cyclase activity of synthesized compounds at the *h*A_2B_AR.

^b^Displacement of specific [3H]-CCPA binding at *h*A_1_AR expressed in Chinese hamster ovary (CHO) cells (*n* = 3–6).

^c^Displacement of specific [3H]-5′-*N*-ethylcarboxamido adenosine (NECA) binding at *h*A_2A_AR expressed in CHO cells (*n* = 3–6).

^d^*K*_i_ values for inhibition of NECA-stimulated adenylyl cyclase activity in CHO cells (*n* = 3–6).

^e^Displacement of specific [3H]-2-(1-hexynyl)-*N*^6^-methyl adenosine (HEMADO) binding at *h*A_3_AR expressed in CHO cells (*n* = 3–6).

## 3. Experimental

### 3.1. Biology

#### 3.1.1. Chinese hamster ovary membranes preparation

The human A_1_, A_2A_, A_2B_, and A_3_ARs were transfected in CHO cells based on the previously reported method [47–50]. The cells were grown adherently and maintained in Dulbecco's modified Eagle's medium with nutrient mixture F12 (DMEM/F12) without nucleosides, containing 10% foetal calf serum, streptomycin (100 µg ml^−1^), penicillin (100 µg ml^−1^), l-glutamine (2 mM) and Geneticin (G418, 0.2 mg ml^−1^) at 37°C in 5% CO_2_, 95% air. The membrane preparation was initiated by removal of culture medium followed by washing of the cells with phosphate buffered saline and scraped off T75 flasks in ice-cold hypotonic buffer (5 mM Tris–HCl, 2 mM EDTA, pH7.4). Then the cell suspension was homogenized with a polytron, and the homogenate was centrifuged for 10 min at 1000 g. The supernatant was then centrifuged for 30 min at 100 000 g. The membrane pellet was suspended in (i) 50 mM Tris–HCl buffer, pH 7.4, for A_1_ARs; (ii) 50 mM Tris–HCl, 10 mM MgCl_2_ buffer, pH 7.4, for A_2A_ARs; and (iii) 50 mM Tris–HCl, 10 mM MgCl_2_, 1 mM EDTA buffer, pH 7.4, for A_3_ARs. The cell suspension was incubated with adenosine deaminase for 30 min at 37°C. Then the membrane preparation was used for binding experiments.

#### 3.1.2. Binding at human A_1_, A_2A_ and A_3_ adenosine receptors

For radioligand binding at A_1_ ARs 1 nM [3H] CCPA was used, whereas 30 nM of [3H] NECA were used for A_2A_ and 10 nM of [3H]- HEMADO were used for A_3_ receptors, respectively [47–50]. Nonspecific binding of [3H]CCPA was determined in the presence of 1 mM theophylline, when [3H]NECA 100 µM R-PIA was used.

#### 3.1.3. Adenylyl cyclase activity

The potency of antagonists at the A_2B_AR was determined in adenylyl cyclase experiments [47–50]. For the measurement of adenylyl cyclase activity, only one high speed centrifugation of the homogenate was used. The resulting crude membrane pellet was resuspended in 50 mM Tris–HCl, pH 7.4 and immediately used for the cyclase assay.

#### 3.1.4. Data analysis

Inhibitory binding constants, *K*_i_, were calculated from the IC_50_ values according to the Cheng and Prusoff equation *K*_i_ = IC_50_/(1 + [C*]/KD*), where [C*] is the concentration of the radioligand and KD* its dissociation constant [49,50]. A weighted nonlinear least-squares curve fitting program LIGAND was also used for computer analysis of inhibition experiments. Potency values (IC_50_) obtained in cyclic AMP assays were calculated by nonlinear regression analysis using the equation for a sigmoid concentration–response curve (Graph Pad Prism, San Diego, CA, USA). All experimental data are expressed as geometric mean with 95% confidence limits in parentheses of three or four independent experiments performed in duplicate.

## 4. Conclusion

In summary, we have designed a class of benzothiazolylquinoline scaffolds for the *h*A_3_ target. Molecular simplification and molecular docking approach using the GLIDE tool from Schrödinger maestro has been employed for the design of these drugs. The effective binding modes of the scaffolds with the receptor binding sites were clearly explained. In addition, we have also performed radioligand binding assay of these scaffolds at *h*A_1_AR, *h*A_2A_AR, *h*A_2B_AR and *h*A_3_AR. We observed that compound **6a** exhibited maximum potency and selectivity in *h*A_3_AR with respect to *h*A_1_AR, *h*A_2A_AR and *h*A_2B_AR which is rationalized with a docking study. Finally, it was concluded that these cytotoxic molecules are selectively targeting to the hA_3_AR.

## Supplementary Material

Discovery of benzothiazolylquinoline conjugates as novel human A3 receptor antagonist: biological evaluations and molecular docking studies; Discovery of benzothiazolylquinoline conjugates as novel human A3 receptor antagonist: biological evaluations and molecular docking studies; Discovery of benzothiazolylquinoline conjugates as novel human A3 receptor antagonist: biological evaluations and molecular docking studies; Discovery of benzothiazolylquinoline conjugates as novel human A3 receptor antagonist: biological evaluations and molecular docking studies

## Data accessibility

Molecular docking images of selected compounds can be found in the electronic supplementary material.
