# Supplementary material for: Discovery of benzothiazolylquinoline conjugates as novel human A3 receptor antagonists: biological evaluations and molecular docking studies
Source: R Soc Open Sci. 2018 Feb 7;5(2):171622. doi: 10.1098/rsos.171622 (PMC5830763; doi:10.1098/rsos.171622)
Supplement: Discovery of benzothiazolylquinoline conjugates as novel human A3 receptor antagonist: biological evaluations and molecular docking studies; Discovery of benzothiazolylquinoline conjugates as novel human A3 receptor antagonist: biological evaluations and molecular docking studies; Discovery of benzo [file rsos171622supp1.docx]

**Supporting Information**

**Discovery of benzothiazolylquinoline conjugates as novel human A_3_ receptor antagonist: biological evaluations and molecular docking studies**

Bidisha Sarkar,^a†^ Santanu Maiti,^a†^ Gajanan Raosaheb Jadhav,^a^ Priyankar Paira,^a^*

Spectroscopic data (^1^H & ^13^C NMR, LCMS, IR) Page 2-7

^1^H & ^13^C NMR, LCMS, IR spectra of compound **3, 4m-o, 6a-l** Page 8-49

X-Ray Crystallographic Data of compound **6c** Page 50

Molecular docking studies Page 51-52

**Conventional synthesis:**

**General procedure for the synthesis of benzothiazolylphenol (3):** 1.59 mmol (200 mg, 170 µL) of aminothiophenol (**1**) and 1.50 mmol (170 µL) of 2-hydroxybenzaldehyde (**2**) was dissolved in methanol and then silica gel was added to it. The mixture was dried to fine powder and kept in a microwave vessel. Subsequently, it was stirred in microwave at 490 W (Temperature 120^ο^ C) for 15 minutes. The progress of reaction was monitored by TLC in 3:1 (hexane: Ethyl acetate). After complete conversion of starting material, the reaction mixture was washed with 2% hexane in Ethyl acetate. Consequently, the solvent was reduced to minimum volume and white needle like crystals of product (2-benzothiazol-2-yl-phenol) (**3**) was obtained with high yield.

**2-benzothiazol-2-yl-phenol (3):** Yield: 82.02%; mp: 78-80°C; R_f_: 0.86; ^1^H NMR (400 MHz, CDCl_3_): δ12.51 (s, 1H, OH), 7.99 (d, J = 7.91 Hz, 1H), 7.88 - 7.92 (m, 1H), 7.69 (dd, J = 1.44, 7.84 Hz, 1H), 7.46 - 7.54 (m, 1H), 7.35 - 7.44 (m, 2H), 7.11 (d, J = 8.4 Hz, 1H), 6.92 - 6.99 (m, 1H); 13C NMR (CDCl3, 125 MHz): δ 115.8 (C), 116.9 (CH), 118.5 (CH), 120.5 (CH), 121.2 (CH), 124.5 (CH), 125.7 (CH), 127.4 (CH), 131.6 (CH), 131.7 (C), 150.9 (C), 156.9 (C), 168.4 (C).

**General procedure for the synthesis of bromoalkoxyphenylbenzothiazole (4m-o):** 1.7 mM (400 mg) of 2-benzothiazole2-ylphenol (**3**) was dissolved in 15 mL acetone and K_2_CO_3_ (1:10) was added to it. Then the solution was refluxed for 30 minutes at 80^ο^C. Dibromo alkanes (2 equivalents) were added to it and the mixture was refluxed for 5 hours. Progress of the reaction was monitored by TLC in hexane: EA (3:1) solvent system. Afterward, the mixture was washed with ethyl acetate followed by warming in water bath for 10 minutes. Finally, Solution was filtered off and the filtrate was evaporated to get the desired product.

**2-[2-(2-bromoethoxy)-phenyl]-benzothiazole (4m):** Yield: 78.67%; mp: 85-88^0^C; R_f_: 0.78; ^1^H NMR (400 MHz, CDCl_3_): δ 8.53 (d, J = 6.0 Hz, 1H), 8.06 (d, J = 8.0 Hz, 1H), 7.92 (d, J = 8.0 Hz, 1H), 7.40 - 7.48 (m, 2H), 7.35 (t, J = 7.2 Hz, 1H), 7.14 (t, J = 7.2 Hz, 1H), 7.00 (d, J = 8.0 Hz, 1H), 4.52 (t, J = 6.4 Hz, 2H, CH_2_), 3.83 (t, J = 6.4 Hz, 2H, CH_2_); 13C NMR (CDCl_3_, 125 MHz): δ 27.4 (CH_2_), 68.1 (CH_2_), 111.6 (CH), 120.3 (CH), 120.9 (CH), 121.7 (CH), 121.9 (C), 123.7 (CH), 124.9 (CH), 128.9 (CH), 130.7 (CH), 135.1(C), 151.1 (C), 154.7 (C), 161.8 (C).

**2-[2-(2-brompropoxy)-phenyl]-benzothiazole (4n):** Yield: 80.27%; mp: 65-68ºC; R_f_: 0.74; ^1^H NMR (400 MHz, CDCl_3_): δ 8.54 (d, J = 7.6 Hz, 1H), 8.10 (d, J = 8.0 Hz, 1H), 7.93 (d, J = 8.0 Hz, 1H), 7.43- 7.51 (m, 2H), 7.38 (t, J = 7.6 Hz, 1H), 7.12 - 7.16 (m, 1H), 7.08 (d, J = 8.4 Hz, 1H), 4.38 (t, J = 5.2 Hz, 2H), 3.78 (t, J = 6.4 Hz, 2H), 2.55 (t, J = 6.0 Hz, 2H); ^13^C NMR (CDCl_3_, 125 MHz) δ 30.2 (CH_2_), 32.3 (CH_2_), 66.7 (CH_2_), 112.4 (C), 121.2 (CH), 121.4 (CH), 122.4 (CH), 122.9 (CH), 124.7 (CH), 126.0 (CH), 129.9 (CH), 131.8 (CH), 135.9 (C), 152.1 (C), 156.3 (C), 162.9 (C).

2-[2-(2-bromobutoxy)-phenyl]-benzothiazole (**4o**): Yield: 90.09%; mp: 70-72ºC; R_f_: 0.72; ^1^H NMR (400 MHz, CDCl_3_): δ 8.54 (d, J = 7.6 Hz, 1H), 8.1 (d, J = 8.4 Hz, 1H), 7.94 (d, J = 8.0 Hz, 1H), 7.46 - 7.51 (m, 1H), 7.43 (d, J = 8.4 Hz, 1H), 7.35-7.39 (m, 1H), 7.14 (t, J = 7.2 Hz, 1H), 7.04 (d, J = 8.0 Hz, 1H), 4.26 (t, J = 5.6 Hz, 2H, CH_2_), 3.56 (t, J = 6.0 Hz, 2H, CH_2_), 2.18 - 2.25 (m, 4H, 2CH_2_); ^13^C NMR (CDCl_3_, 125 MHz): δ 26.8 (CH_2_), 28.5 (CH_2_), 32.4 (CH_2_), 67.1 (CH_2_), 111.1 (CH), 120.1 (CH), 120.2 (CH), 121.3 (CH), 121.8 (C), 123.6 (CH), 124.9 (CH), 128.7 (CH), 130.7 (CH), 135.0 (C), 151.1 (C), 155.4 (C), 161.9 (C).

**Conventional method for the preparation of 8-[2-(2-benzothiazol-2-yl-phenoxy)-ethoxy]-quinoline (6a):** 0.1 mmol (50 mg) of 2-[2-(2-bromoethoxy)-phenyl]-benzothiazole (**4m**) and 8-hydroxyquinoline (**5a**) (1:1) were dissolved in DMF and then K_2_CO_3_ (1:10) was added to it. Reaction mixture was kept in microwave vessel followed by stirring for 10 min at 350 W (Temperature 100^ο^ C) under the microwave. Progress of the reaction was monitored by TLC using hexane: ethyl acetate solvent system (3:1). The reaction mixture was extracted with ethyl acetate. Organic layer was separated and aqueous layer was further extracted with ethyl acetate (3x10 ml). The volume of ethyl acetate was reduced to run silica gel column chromatography for isolation of pure compounds.

**Green procedure for the Synthesis of 8-[2-(2-benzothiazol-2-yl-phenoxy)-alkoxy]-quinoline (6a-l) in one pot three step sequence:** Equivalent amount of 2-aminothiophenol (**1**) and 2-hydroxybenzaldehyde (**2**) was mixed together in a microwave vessel followed by stirring under the microwave at 490 watt (Temperature 120^ο^ C) for 30 minutes. Formation of first step product was confirmed by TLC. The resulting mixture was further treated with excess dibromoalkanes (2 equivalents) in presence of catalytic amount of amberlite IRA-402(OH) under microwave at 350 watt (Temperature 100^ο^ C) for 10 minutes. Formation of alkylated product was also confirmed by TLC. Afterwards, 1 equivalent of 8-hydroxyquinoline derivatives (**5a-d**) was added to the reaction mixtures and stirring continued in the microwave for another 10 minutes. The progress of the reaction was monitored by TLC. After completion of the reaction, microwave vessel was allowed to cool for some time. Then, the mixture was treated with ethylacetate and the resin was filtered off. Subsequently, the solvent ethylacetate was evaporated to optimum volume to run silica gel column chromatography for the isolation of pure compounds. White needle like crystals of 2-(2′hydroxyphenyl)benzothiazolylquinoline (**6a-l**) was obtained by column purification with high yield.

**8-[2-(2-benzothiazol-2-yl-phenoxy)-ethoxy]-quinoline (6a):** Yield: 92.5 %; mp: 94-97ºC; R_f_: 0.92 (75% ethyl acetate in hexane); λmax (H_2_O, nm) 293; ^1^H NMR (400 MHz, CDCl3): δ 8.91 - 8.93 (m, 1H), 8.52 (d, J = 8.0 Hz, 1H), 8.15 (d, J = 8.0 Hz, 1H), 7.99 – 8.05 (m, 2H), 7.66 (d, J = 8.0 Hz, 1H), 7.43 - 7.49 (m, 5H), 7.12 - 7.25 (m, 3H), 4.79 – 4.83 (m, 4H); ^13^C NMR (125 MHz, CDCl3): δ 66.1 (CH_2_), 66.4 (CH_2_), 108.8 (CH), 111.7 (CH), 119.5 (CH), 120.1 (CH), 120.6 (CH), 120.7 (CH), 121.6 (C), 121.7 (CH), 123.5 (CH), 124.8 (CH), 125.6 (CH), 128.6 (CH), 128.7 (CH), 130.8 (CH), 134.9 (C), 135.1 (C), 148.4 (CH), 151.1 (C), 152.0 (C), 155.2 (C), 161.5 (C), 162.2 (C); IR (KBr, cm-1): 3061, 2773, 2337, 2914, 1500, 1597, 1379; ESI-MS m/z = 399 [M+H]^+^; HRMS: m/z calcd. for C_24_H_18_N_2_O_2_S+H^+^: 399.1167; found 399.1170. Purity of the complex was determined by elemental analysis. Anal.Calcd for C_24_H_18_N_2_O_2_S (%): C 72.34, H 4.55, N 7.03. found: C 72.05, H 4.08, N 6.92

**8-[2-(2-benzothiazol-2-yl-phenoxy)-ethoxy]-5,7-dibromoquinoline**(**6b**):Yield: 88.56%; mp: 110-112ºC; R_f_: 0.84 (10% ethyl acetate in hexane); λmax (H_2_O, nm) 306; ^1^H NMR (400 MHz, CDCl3): δ 8.89 (d, J = 5.2 Hz, 1H), 8.46 (d, J = 9.6 Hz, 1H), 8.38 (d, J = 10.0 Hz, 1H), 8.05 (d, J = 8.0 Hz, 1H), 7.84 (s, 1H), 7.70 (d, J = 7.6 Hz, 1H), 7.42 - 7.51 (m, 3H), 7.32 - 7.36 (m, 1H), 7.10 - 7.14 (m, 2H), 5.06 (t, J = 4.8 Hz, 2H), 4.73 (t, J = 4.4 Hz, 2H); ^13^C NMR (125 MHz, CDCl3): δ 67.81 (CH_2_), 72.02 (CH_2_), 111.3 (CH), 115.3 (C), 115.6 (C), 120.1 (CH), 120.3 (CH), 121.3 (CH), 121.4 (CH), 121.7 (CH), 123.5 (C), 124.7 (CH), 127.0 (CH), 128.6 (CH), 130.7 (CH), 132.4 (CH), 134.9 (C), 135.0 (C), 142.5 (C), 149.5 (CH), 151.0 (C), 151.6 (C), 155.2 (C), 162.0 (C); IR (KBr, Cm^-1^): 3066, 1597, 1575, 1450; ESI-MS m/z = 557 [M+H]^+^, HRMS: m/z calcd. for C_24_H_16_Br_2_N_2_O_2_S+H^+^: 556.9357; found 556.9352. Purity of the complex was determined by elemental analysis. Anal. Calcd for C_24_H_16_Br_2_N_2_O_2_S (%): C 51.82, H 2.90, N 5.04. found: C 51.55, H 2.98, N 5.32

**8-[2-(2-benzothiazol-2-yl-phenoxy)-ethoxy]-5-chloroquinoline (6c):** Yield: 84.87%; mp: 92-95ºC; R_f_: 0.42 (25% ethyl acetate in hexane); λmax (H_2_O, nm); ^1^H NMR (400 MHz, CDCl_3_): δ 8.97 (d, J = 5.2 Hz, 1H), 8.49 - 8.55 (m, 2H), 8.05 (d, J = 8.4 Hz 1H) 7.66 (d, J = 7.2 Hz, 1H), 7.51 - 7.55 (m, 2H), 7.43 - 7.48 (m, 2H), 7.31 (t, J = 7.2 Hz, 1H), 7.15 - 7.17 (m, 3H), 4.78 – 4.82 (m, 4H, CH2); ^13^C NMR (125 MHz, CDCl_3_): δ 66.3 (CH_2_), 66.55 (CH_2_), 108.8 (C), 111.7 (CH), 120.0 (CH), 120.7 (CH), 121.4 (CH), 121.6 (C), 121.7 (CH), 122.1 (CH), 123.5 (C), 124.8 (CH), 125.3 (CH), 126.2 (CH), 128.7 (CH), 130.8 (CH), 132.0 (CH), 135.0 (C), 139.9 (C), 148.9 (CH), 151.1 (C), 152.7 (C), 155.1 (C), 162.1 (C); IR (KBr, cm^-1^): 3093, 3053, 2916, 2866, 1593, 435; ESI-MS m/z = 433 [M+H]^+^, 435 [M+2+H]^+^; HRMS: m/z calcd. For C_24_H_17_ClN_2_O_2_S+H^+^: 433.0778; found 433.0783. Purity of the complex was determined by elemental analysis. Anal. Calcd for C_24_H_17_ClN_2_O_2_S (%): C 66.58, H 3.96, N 6.47. found: C 66.35, H 3.48, N 6.22

**8-[2-(2-benzothiazol-2-yl-phenoxy)-ethoxy]-5-chloro-7-iodoquinoline (6d):** Yield: 83.6%; mp: 125-127ºC; R_f_: 0.34 (75% ethyl acetate in hexane); λmax (H_2_O, nm) 314; ^1^H NMR (400 MHz, CDCl_3_): δ 8.92 (d, J = 5.2 Hz, 1H), 8.44 - 8.48 (m, 2H), 8.05 (d, J = 8.0 Hz, 1H), 7.82 (s, 1H), 7.71 (d, J = 8.0 Hz, 1H), 7.52 (dd, J = 4.4, 8.4 Hz, 1H), 7.46 (t, J = 8.0 Hz, 2H), 7.34 (t, J = 7.6 Hz, 1H), 7.10 -7.15 (m, 2H), 5.06 (t, J = 4.8 Hz, 2H), 4.76 (t, J = 4.8 Hz, 2H); ^13^C NMR (125 MHz, CDCl_3_): δ 68.8 (CH_2_), 73.2 (CH_2_), 90.1 (C), 112.4 (CH), 120.6 (CH), 121.1 (CH), 121.3 (C), 122.3 (CH), 122.7 (C), 124.5 (C), 125.8 (CH), 126.5 (CH), 127.5 (CH), 128.6 (CH), 131.7 (CH), 133.5 (CH), 135.0 (CH), 136.1 (C), 142.1 (C), 150.3 (CH), 152.0 (C), 155.2 (C), 156.3 (C), 163.2 (C); IR (KBr, cm^-1^): 3053, 2945, 2924, 2850, 1570, 1498; ESI-MS m/z = 559 [M+H]^+^, 561 [M+2+H]^+^; HRMS: m/z calcd. for C_24_H_16_ClIN_2_O_2_S+H^+^: 558.9744; found 558.9746. Purity of the complex was determined by elemental analysis. Anal.Calcd for C_24_H_16_ClIN_2_O_2_S (%): C 51.58, H 2.89, N 5.01. found: C 51.25, H 2.68, N 4.88

**2-(2-(3-(quinolin-8-yloxy)propoxy)phenyl)benzo[d]thiazole (6e):** Yield: 84.1%; mp: 108-110ºC; R_f_: 0.88 (75% ethyl acetate in hexane); λmax (H2O, nm) 313; ^1^H NMR (400 MHz, CDCl_3_): δ 8.96 (brs, 1H), 8.53 (d, J = 6.9 Hz, 1H), 8.07 – 8.13 (m, 2H), 7.86 (d, J = 8.0 Hz, 1H), 7.36 - 7.49 (m, 6H), 7.09 - 7.12 (m, 3H), 4.60 - 4.63 (m, 2H), 4.52- 4.55 (m, 2H), 2.75 (m, 2H); ^13^C NMR(125 MHz, CDCl_3_): δ 28.2 (CH2), 64.9 (CH2), 65.1 (CH2), 108.1 (CH), 111.4 (CH), 118.8 (CH), 120.1 (CH), 120.2 (CH), 120.6 (CH), 121.2 (C), 121.8 (CH), 123.6 (CH), 124.9 (CH), 125.0 (CH), 125.7 (CH), 128.5 (CH), 128.6 (C), 130.8 (CH), 134.9 (C), 139.4 (C), 148.3 (CH), 151.1 (C), 153.6 (C), 155.5 (C), 162.0 (C); IR (KBr, cm-1): 3037, 2924, 1587, 1527; ESI-MS m/z = 413 [M+H]^+^; HRMS: m/z calcd. for C_25_H_20_N_2_O_2_S+H^+^: 413.1324; found 413.1320. Purity of the complex was determined by elemental analysis. Anal.Calcd for C_25_H_20_N_2_O_2_S (%): C 72.79, H 4.89, N 6.79. found: C 72.55, H 4.78, N 6.52

**2-(2-(3-((5,7-dibromoquinolin-8-yl)oxy)propoxy)phenyl)benzo[d]thiazole (6f):** Yield: 80.98%; mp: 102-104ºC; R_f_: 0.39 (10% ethyl acetate in hexane); λmax (H_2_O, nm) 269; ^1^H NMR (400 MHz, CDCl_3_): δ 8.73 (br s, 1H), 8.54 (d, J = 7.6 Hz, 1H), 8.42 (d, J = 8.4 Hz, 1H), 8.00 - 8.06 (m, 1H), 7.94 (s, 1H), 7.83 (d, J = 8.0 Hz, 1H), 7.44 - 7.47 (m, 3H), 7.29 - 7.35 (m, 1H), 7.11 - 7.18 (m, 2H), 4.73 (t, J = 6.0 Hz, 2H), 4.66 (t, J = 5.6 Hz, 2H), 2.64 – 2.67 (m, 2H); ^13^C NMR (125 MHz, CDCl_3_): δ 29.9 (CH_2_), 64.9 (CH_2_), 70.9 (CH_2_), 111.3 (CH), 115.3 (C), 115.4 (C), 119.9 (CH), 120.1 (CH), 121.1 (CH), 121.5 (CH), 121.7 (CH), 123.5 (CH), 124.8 (CH), 127.1 (C), 128.5 (CH), 130.8 (CH), 132.5 (CH), 134.9 (C), 135.0 (C), 142.7 (C), 149.6 (CH), 151.0 (C), 151.6 (C), 155.7 (C), 162.2 (C); IR (KBr, cm^-1^): 3055, 2335, 1573; ESI-MS m/z = 571 [M+H] ^+^, 573 [M+2+H] ^+^; HRMS: m/z calcd. for C_25_H_18_Br_2_N_2_O_2_S+H^+^: 570.9514; found 570.9518. Purity of the complex was determined by elemental analysis. Anal.Calcd for C_25_H_18_Br_2_N_2_O_2_S (%): C 52.65, H 3.18, N 4.91. found: C 52.35, H 2.98, N 4.52

**2-(2-(3-((5-chloroquinolin-8-yl)oxy)propoxy)phenyl)benzo[d]thiazole (6g):** Yield: 90.39%; mp: 80-82ºC; R_f_: 0.36 (25% ethyl acetate in hexane); λmax (H_2_O, nm) 316; ^1^H NMR (400 MHz, CDCl_3_): δ 9.00 (br s, 1H), 8.52 (t, J = 6.0 Hz, 2H), 8.08 (d, J = 8.0 Hz, 1H), 7.87 (d, J = 7.6 Hz, 1H), 7.53 - 7.56 (m, 1H), 7.34-7.50 (m, 4H), 7.08 - 7.13 (m, 2H), 7.01 (d, J = 8.4 Hz, 1H), 4.59 (t, J = 6.0 Hz, 2H), 4.52 (t, J = 6.0 Hz, 2H), 2.72 – 2.75 (m, 2H); ^13^C NMR (125 MHz, CDCl_3_): δ 28.1 (CH_2_), 64.9 (CH_2_), 65.2 (CH_2_), 107.9 (C), 111.4 (CH), 120.1 (CH), 120.2 (CH), 121.2 (C), 121.3 (CH), 121.4 (CH), 121.8 (CH), 123.6 (CH), 124.9 (CH), 125.4 (CH), 126.1 (C), 128.7 (CH), 130.8 (CH), 132.0 (CH), 134.9 (C), 139.9 (C), 148.8 (CH), 151.1 (C), 152.9 (C), 155.4 (C), 161.9 (C); IR (KBr, cm^-1^): 2916, 2850, 1573; ESI-MS m/z = 447 [M+H] ^+^, 449 [M+2+H] ^+^; HRMS: m/z calcd. for C_25_H_19_ClN_2_O_2_S+H^+^: 447.0934; found 447.0939. Purity of the complex was determined by elemental analysis. Anal.Calcd for C_25_H_19_ClN_2_O_2_S (%): C 67.18, H 4.28, N 6.27. found: C 66.75, H 3.98, N 6.12

**2-(2-(3-((5-chloro-7-iodoquinolin-8-yl)oxy)propoxy)phenyl)benzo[d]thiazole (6h):** Yield: 84.6%; mp: 138-140ºC; R_f_: 0.91 (75% ethyl acetate in hexane); λmax (H_2_O, nm) 330; ^1^H NMR (400 MHz, CDCl_3_): δ 8.73 (d, J = 5.2 Hz, 1H), 8.55 (d, J = 9.6 Hz, 1H), 8.47 (d, J = 8.8 Hz, 1H), 8.07 (d, J = 8.4 Hz, 1H), 7.93 (s, 1H), 7.85 (d, J = 8.0 Hz, 1H), 7.44 - 7.48 (m, 3H), 7.33 - 7.36 (m, 1H), 7.11 - 7.19 (m, 2H), 4.60 -4.74 (m, 4H), 2.68 (t, J = 6.0 Hz, 2H); ^13^C NMR (125 MHz, CDCl_3_): δ 29.3 (CH_2_), 65.0 (CH_2_), 70.8 (CH_2_), 89.3 (C), 111.3 (CH), 119.9 (CH), 120.2 (CH), 121.1 (C), 121.3 (CH), 121.7 (CH), 123.4 (CH), 124.8 (CH), 125.6 (C), 126.5 (C), 128.5 (CH), 130.8 (CH), 132.4 (CH), 133.9 (CH), 135.0 (C), 141.4 (C), 149.3(CH), 151.2 (C), 154.1 (C), 155.7 (C), 162.2 (C); IR (KBr, cm^-1^): 3059, 2920, 1688, 1598, 1585; ESI-MS m/z = 573 [M+H]^+^, 575 [M+2+H] ^+^; HRMS: m/z calcd. for C_25_H_18_ClIN_2_O_2_S+H^+^: 572.9900; found 572.9905. Purity of the complex was determined by elemental analysis. Anal.Calcd for C_25_H_18_ClIN_2_O_2_S (%): C 52.42, H 3.17, N 4.89. found: C 44.75, H 3.98, N 6.32

**2-(2-(4-(quinolin-8-yloxy)butoxy)phenyl)benzo[d]thiazole (6i):** Yield: 87.3%; mp: 120-123ºC; R_f_: 0.56 (75% ethyl acetate in hexane); λmax (H_2_O, nm) 332; ^1^H NMR (400 MHz, CDCl3): δ 8.94 (d, J = 6.0 Hz, 1H), 8.53 (d, J = 9.2 Hz, 1H), 8.06 - 8.13 (m, 2H), 7.88 (d, J = 7.6 Hz, 1H), 7.33 - 7.49 (m, 6H), 7.06 - 7.12 (m, 3H), 4.33 - 4.44 (m, 4H), 2.35 (brs, 4H); 13C NMR (125 MHz, CDCl_3_): δ 24.9 (CH_2_), 25.3 (CH_2_), 67.5 (CH_2_), 67.9 (CH_2_), 107.8 (CH), 111.3 (CH), 118.6 (CH), 119.9 (CH), 120.2 (CH), 120.6 (CH), 121.2 (CH), 121.7 (C), 123.5 (CH), 124.8 (CH), 125.6 (CH), 128.5 (CH), 128.6 (CH), 130.7 (CH), 134.7 (CH), 134.9 (C), 135.1 (C), 139.4 (C), 148.3 (CH), 151.1 (C), 153.7 (C), 155.6 (C), 162.1 (C); IR (KBr, cm^-1^): 3052, 2912, 2872, 2330; ESI-MS m/z = 427 [M+H] ^+^; HRMS: m/z calcd. for C_26_H_22_N_2_O_2_S+H^+^: 427.1480; found 427.1484. Purity of the complex was determined by elemental analysis. Anal.Calcd for C_26_H_22_N_2_O_2_S (%): C 73.21, H 5.20, N 6.57. found: C 72.85, H 4.98, N 6.22

**2-(2-(4-((5,7-dibromoquinolin-8-yl)oxy)butoxy)phenyl)benzo[d]thiazole (6j):** Yield: 82.56%; mp: 112-114ºC; R_f_ : 0.49 (10% ethyl acetate in hexane); λmax (H_2_O, nm) 318; ^1^H NMR (400 MHz, CDCl_3_): δ 8.90 (d, J = 5.6 Hz, 1H), 8.55 (dd, J = 9.2 Hz, 1H), 8.46 (d, J = 10.0 Hz, 1H), 8.08 (d, J = 8.0 Hz, 1H), 7.98 (s, 1H), 7.89 (d, J = 8.0 Hz, 1H), 7.42 - 7.51 (m, 3H), 7.32 - 7.38 (m, 1H), 7.09 - 7.14 (m, 2H), 4.52 (t, J = 6.0 Hz, 2H), 4.41 (t, J = 6.0 Hz, 2H), 2.38 - 2.41 (m, 2H), 2.27 - 2.30 (m, 2H); ^13^C NMR (125 MHz, CDCl_3_): δ 26.1 (CH_2_), 27.3 (CH_2_), 68.9 (CH_2_), 74.8 (CH_2_), 112.3 (CH), 116.1 (C), 116.5 (C), 121.0 (CH), 121.2 (CH), 122.3 (CH), 122.5 (CH), 122.8 (CH), 124.5 (CH), 125.7 (CH), 128.1 (C), 129.6 (CH), 131.7 (CH), 133.6 (CH), 136.0 (C), 136.3 (C), 141.2 (C), 150.6 (CH), 152.1 (C), 152.8 (C), 156.7 (C), 163.2 (C); IR (KBr, cm^-1^): 3051, 2939, 2875, 1595, 1575; ESI-MS m/z = 585 [M+H] ^+^, 587 [M+2+H]^+^; HRMS: m/z calcd. for C_26_H_20_Br_2_N_2_O_2_S+H^+^: 584.9670; found 584.9674. Purity of the complex was determined by elemental analysis. Anal.Calcd for C_26_H_20_Br_2_N_2_O_2_S (%): C 53.44, H 3.45, N 4.79. found: C 53.15, H 3.28, N 4.62

**2-(2-(4-((5-chloroquinolin-8-yl)oxy)butoxy)phenyl)benzo[d]thiazole (6k):** Yield: 86.04%; mp: 110-112ºC; R_f_: 0.86 (25% ethyl acetate in hexane); λmax (H_2_O, nm) 324; ^1^H NMR (400 MHz, CDCl_3_): δ 8.97 (d, J = 5.2 Hz, 1H), 8.50 - 8.54 (m, 2H), 8.08 (d, J = 8.0 Hz, 1H), 7.86 (d, J = 7.6 Hz, 1H), 7.34 - 7.55 (m, 5H), 7.06 - 7.12 (m, 2H), 6.96 (d, J = 8.40 Hz, 1H), 4.36 - 4.37 (m, 4H), 2.22 - 2.33 (m, 4H); ^13^C NMR (CDCl_3_, 125 MHz): δ 24.9 (CH_2_), 25.2 (CH_2_), 67.7 (CH_2_), 67.8 (CH_2_), 107.6 (C), 111.3 (CH), 120.0 (CH), 120.2 (CH), 121.1 (CH), 121.2 (CH), 121.3 (CH), 121.7 (C), 123.5 (CH), 124.9 (CH), 125.4 (CH), 126.1 (C), 128.6 (CH), 130.7 (CH), 132.0 (CH), 135.0 (C), 139.9 (C), 148.7 (CH), 151.1 (C), 152.8 (C), 155.5 (C), 162.1 (C); IR (KBr, cm-1): 3064, 2941, 2873, 1598, 1585, 1566; ESI-MS m/z = 461 [M+H] ^+^, 463 [M+2+H] ^+^; HRMS: m/z calcd. for C_26_H_21_ClN_2_O_2_S+H+: 461.1091; found 461.1094. Purity of the complex was determined by elemental analysis. Anal.Calcd for C_26_H_21_ClN_2_O_2_S (%): C 67.74, H 4.59, N 6.08. found: C 67.65, H 4.28, N 5.82

**2-(2-(4-((5-chloro-7-iodoquinolin-8-yl)oxy)butoxy)phenyl)benzo[d]thiazole (6l):** Yield: 82.3%; mp: 105-108ºC; R_f_: 0.90 (75% ethyl acetate in hexane);λmax (H_2_O, nm) 264; ^1^H NMR (400 MHz, CDCl3): δ 8.90 (d, J = 5.2 Hz, 1H), 8.48 - 8.56 (m, 2H), 8.08 (d, J = 8.0 Hz, 1H), 7.96 (s, 1H), 7.90 (d, J = 8.0 Hz, 1H), 7.42 - 7.52 (m, 3H), 7.36 (t, J = 7.6 Hz, 1H), 7.10 - 7.14 (m, 2H), 4.51 (t, J = 6.0 Hz, 2H), 4.43 (t, J = 6.0 Hz, 2H), 2.30 - 2.33 (m, 2H), 2.39 - 2.43 (m, 2H); ^13^C NMR (125 MHz, CDCl_3_): δ 25.1 (CH_2_), 26.3 (CH_2_), 67.9 (CH_2_), 73.7 (CH_2_), 89.3 (C), 111.3 (CH), 119.9 (CH), 120.2 (CH), 121.2 (CH), 121.7 (CH), 121.8 (CH), 123.5 (CH), 124.8 (CH), 125.3 (C), 126.5 (C), 128.6 (C), 130.7 (CH), 132.5 (CH), 134.1 (CH), 135.1 (C), 141.4 (C), 149.3 (CH), 151.1 (C), 154.3 (C), 155.7 (C), 162.2 (C); IR (KBr, cm^-1^): 2939, 1595, 1573; ESI-MS m/z = 587 [M+H] ^+^, 589 [M+2+H] ^+^; HRMS: m/z calcd. for C_26_H_20_ClIN_2_O_2_S+H^+^: 587.0057; found 587.0059. Purity of the complex was determined by elemental analysis. Anal.Calcd for C_26_H_20_ClIN_2_O_2_S (%): C 53.21, H 3.43, N 4.77. found: C 52.85, H 3.18, N 4.52

^1^H NMR of compound **3**


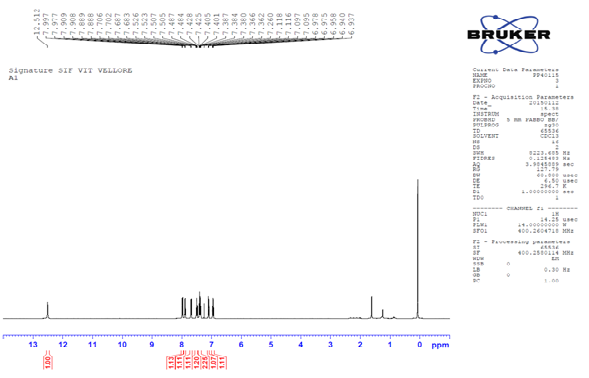

^13^C NMR of compound **3**


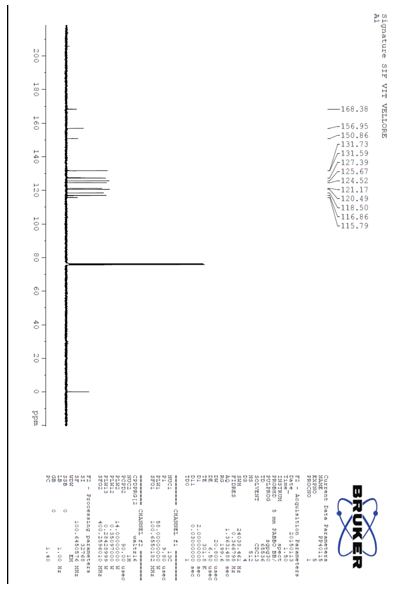

^1^H NMR of compound **4m**

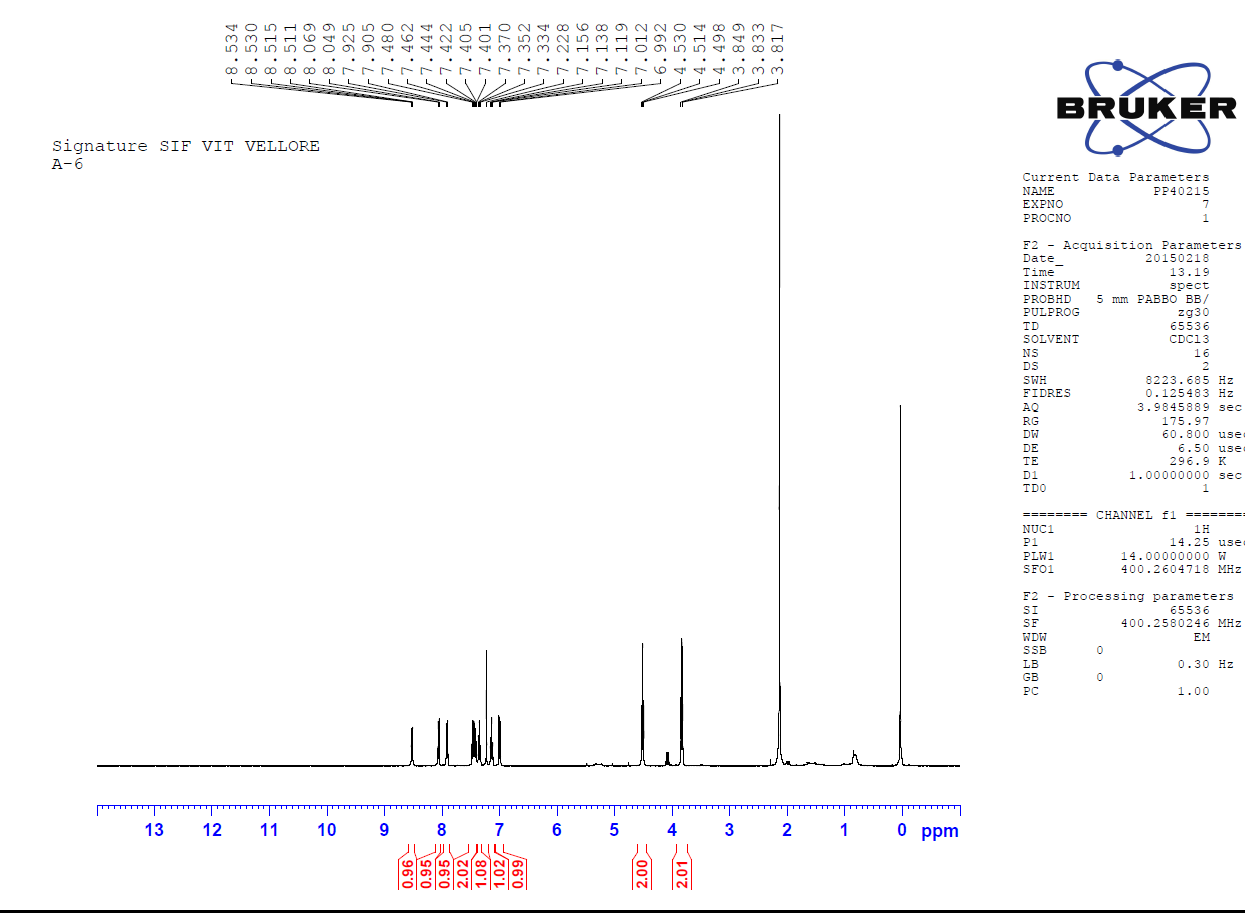

^13^C NMR of compound **4m**


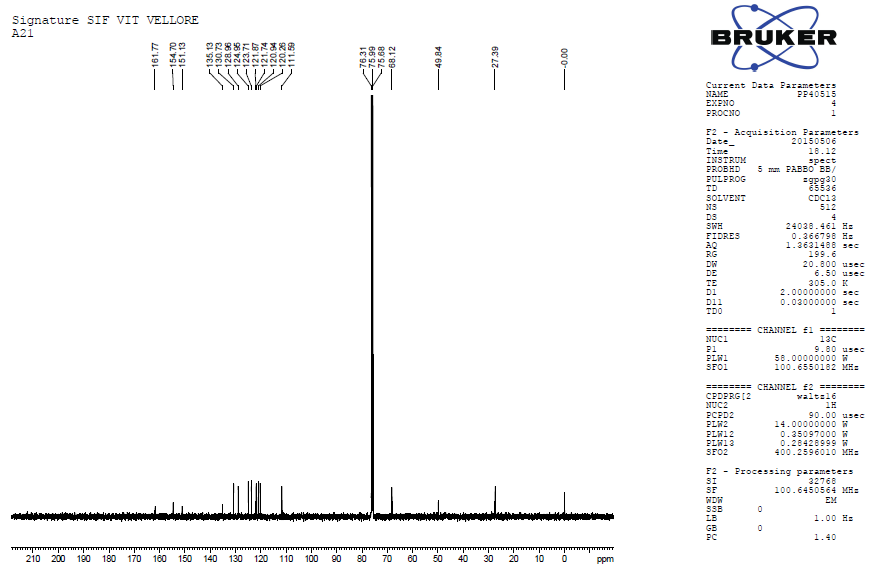

^1^H NMR of compound **4n**

**
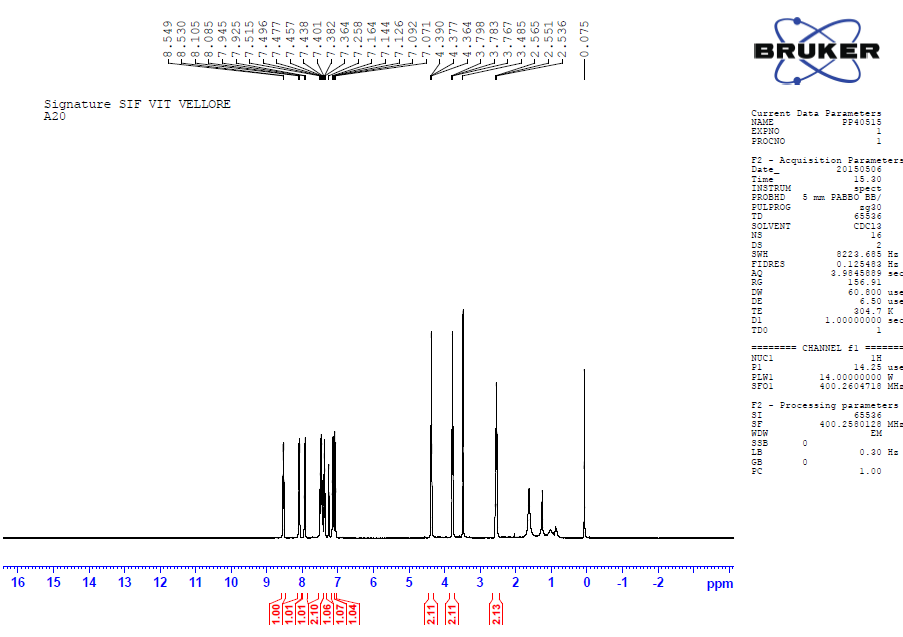
**

**
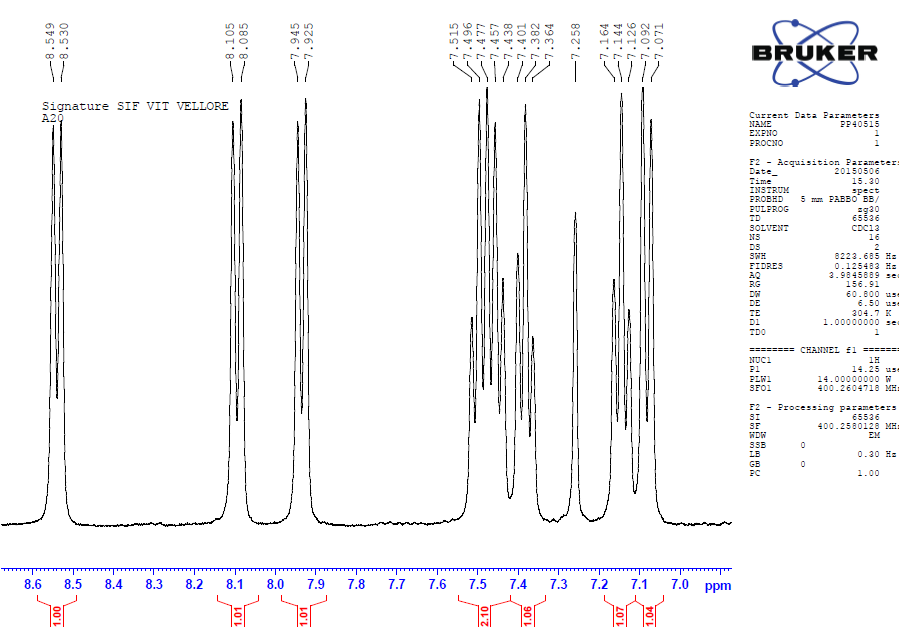
**

^13^C NMR of compound **4n**


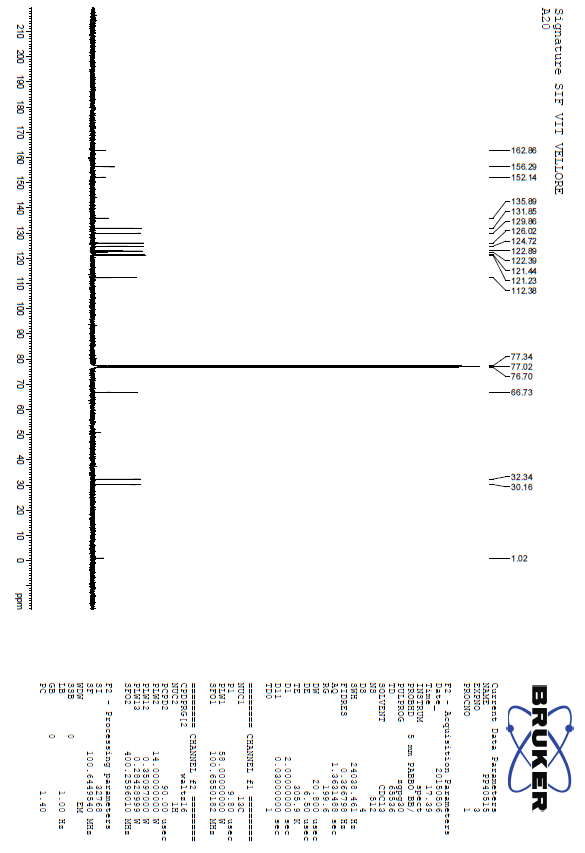

^1^H NMR of compound **4o**

^13^C NMR of compound **4o**


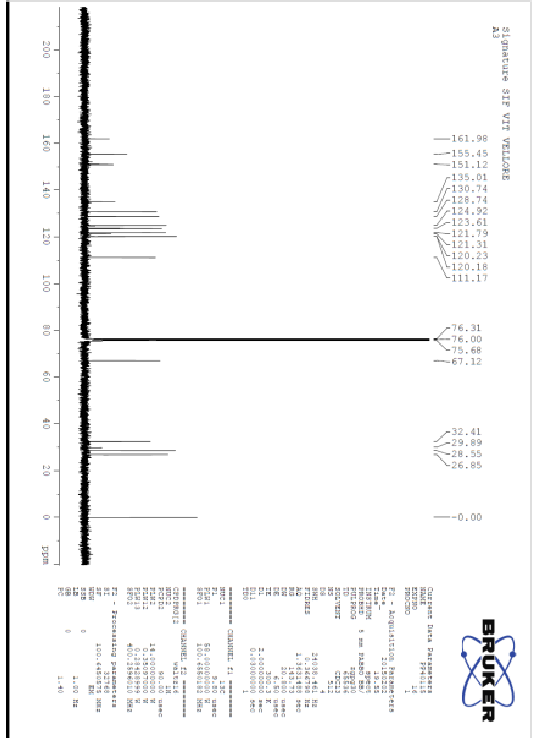

^1^H NMR of compound **6a**

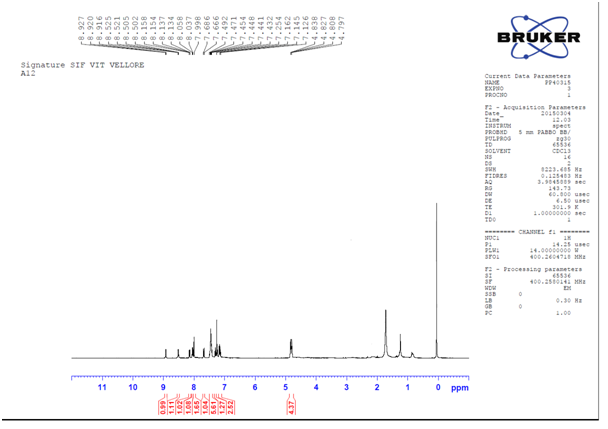

^13^C NMR of compound **6a**


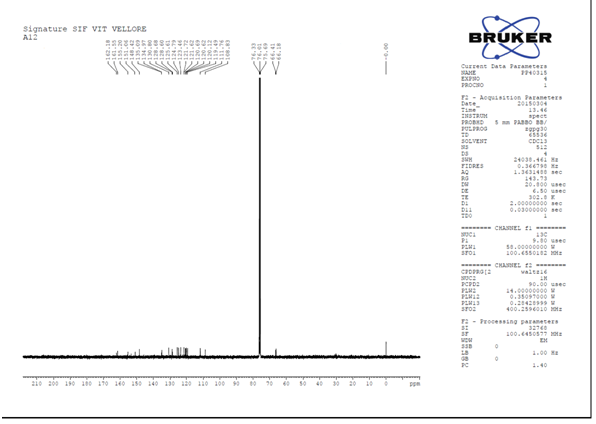

LC-MS of compound **6a**

IR of compound **6a**

^1^H NMR of compound **6b**


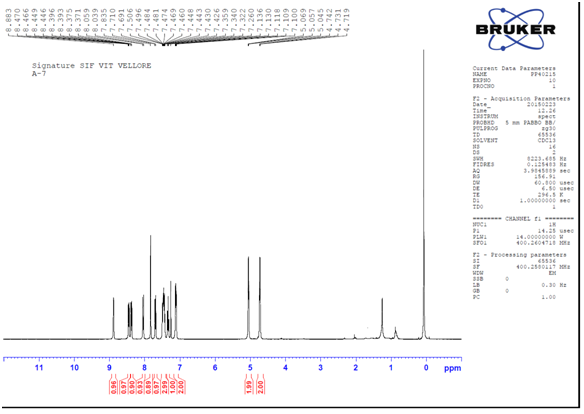

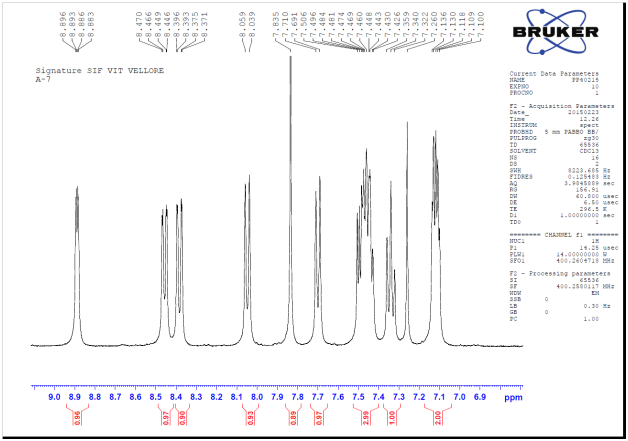


^13^C NMR of compound **6b**


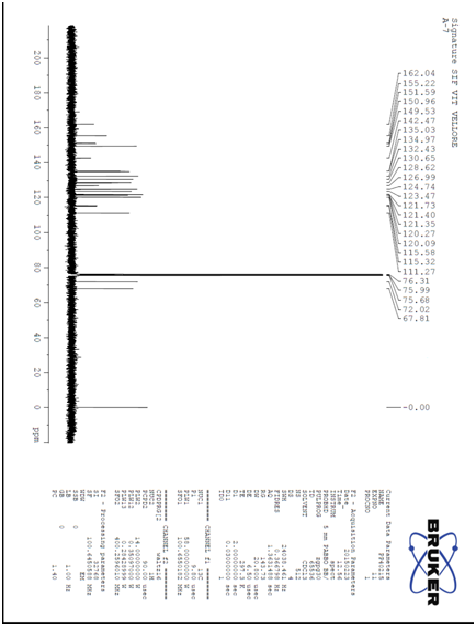

LC-MS of compound **6b**

IR of compound **6b**

^1^H NMR of compound **6c**


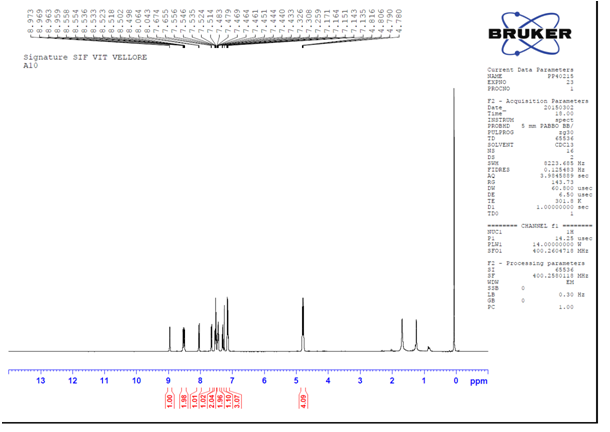

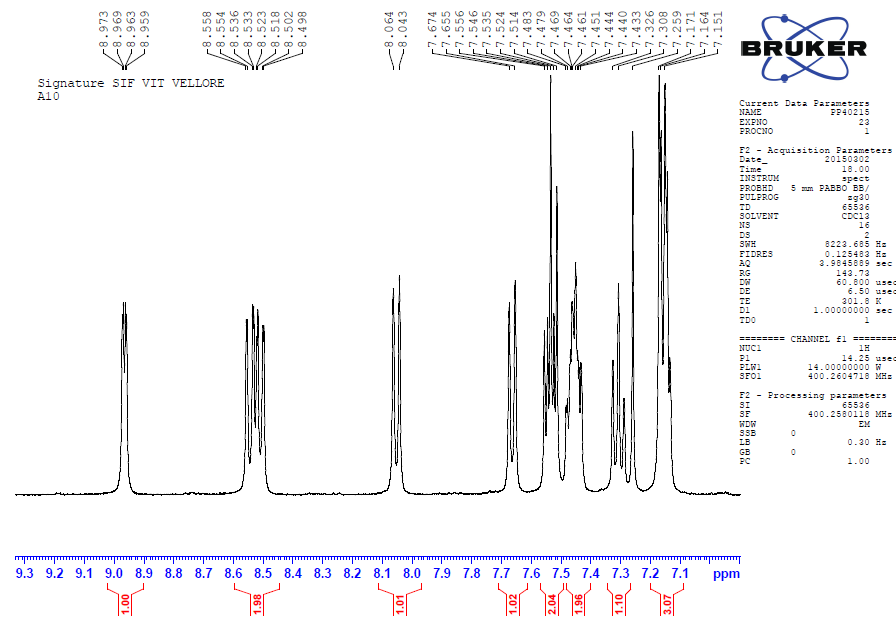


^13^C NMR of compound **6c**


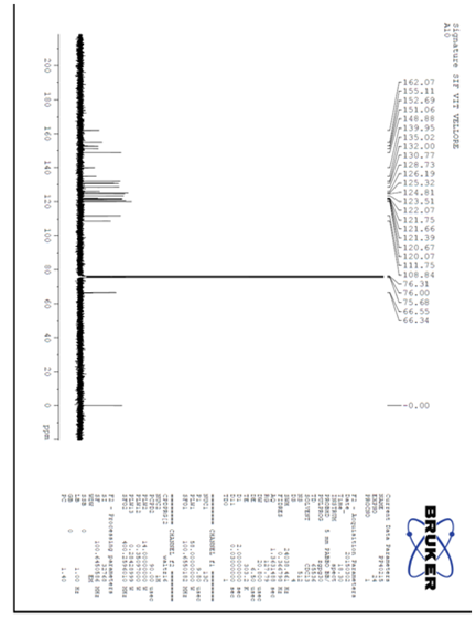

IR of compound **6c**

^1^H NMR of compound **6d**


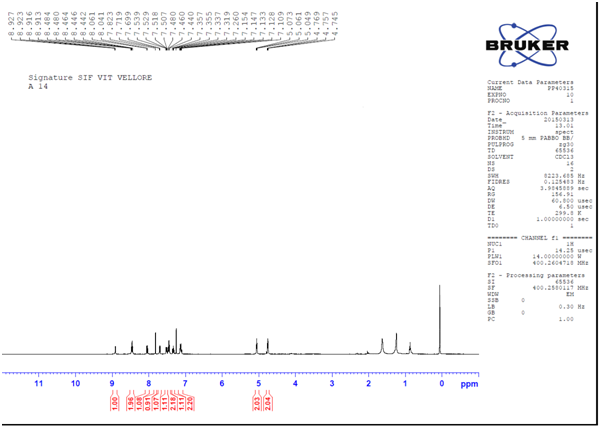

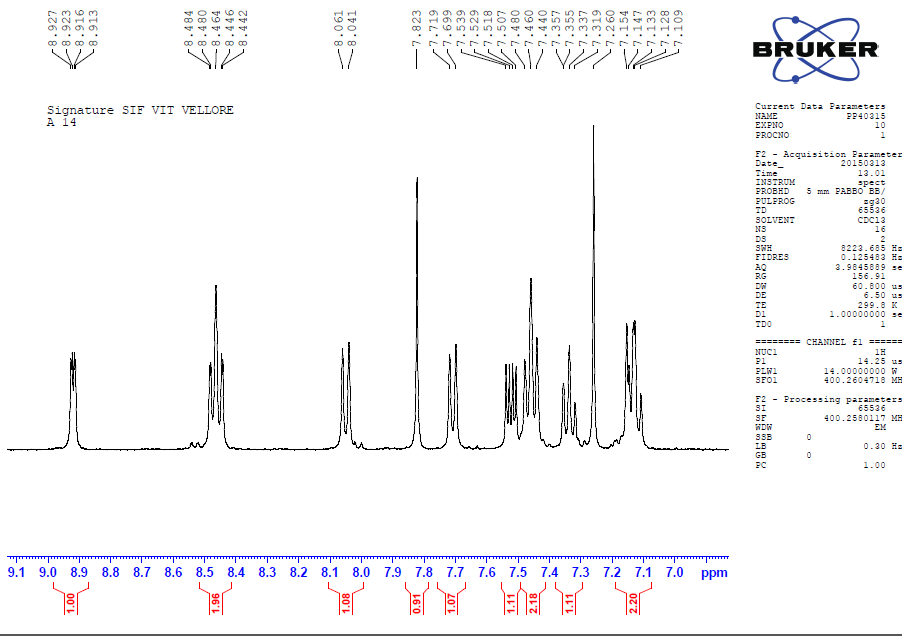


^13^C NMR of compound **6d**


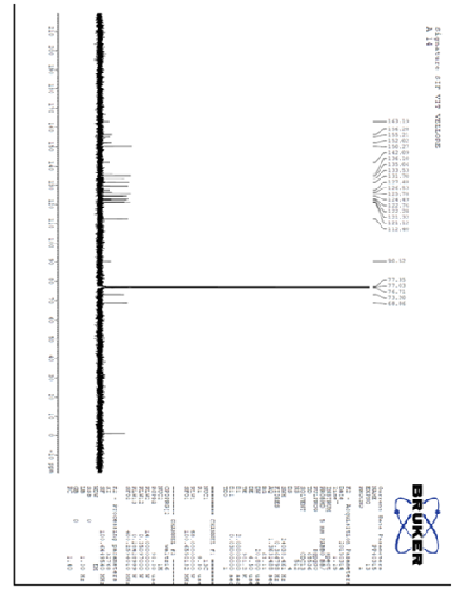

IR of compound **6d**

^1^H NMR of compound **6e**


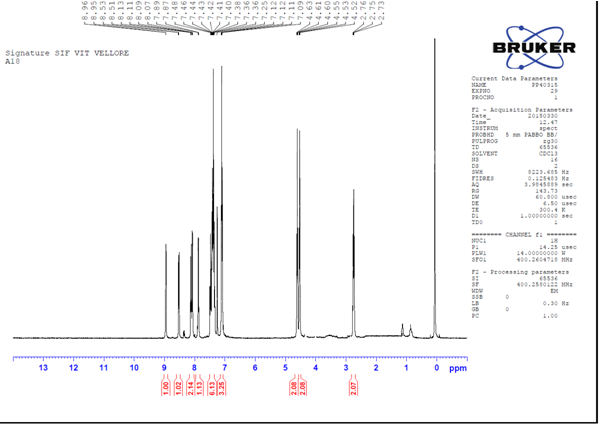

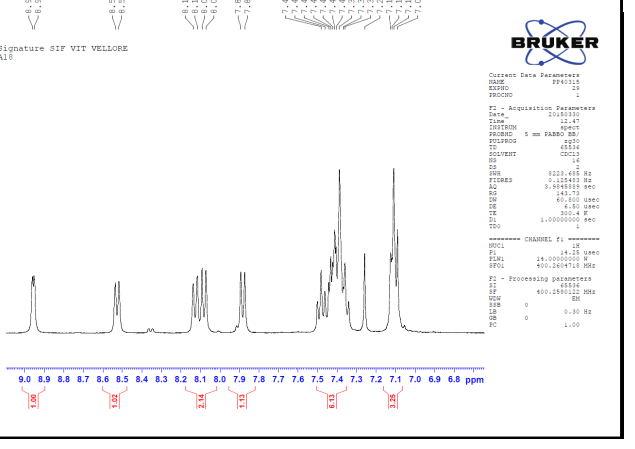


^13^C NMR of compound 6e


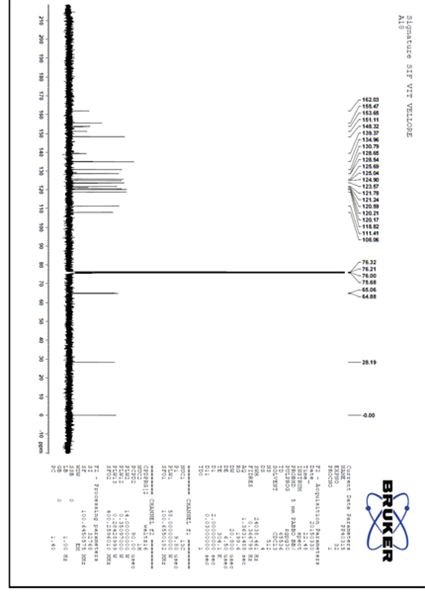

IR of compound **6e**

^1^H NMR of compound **6f**


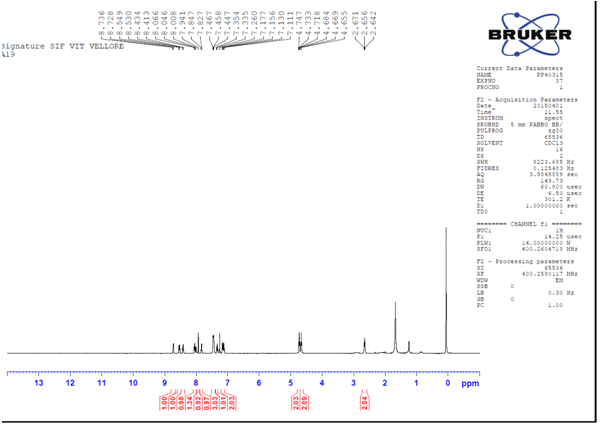

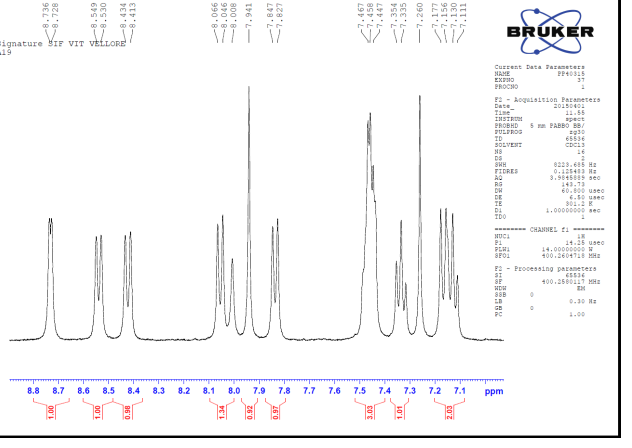


^13^C NMR of compound **6f**


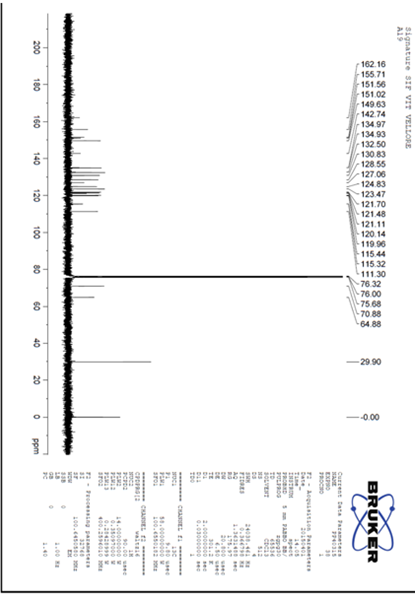

LC-MS of compound **6f**

IR of compound **6f**

^1^H NMR of compound **6g**


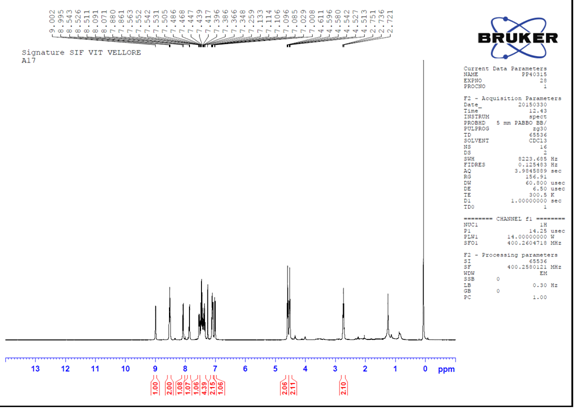

^13^C NMR of compound **6g**


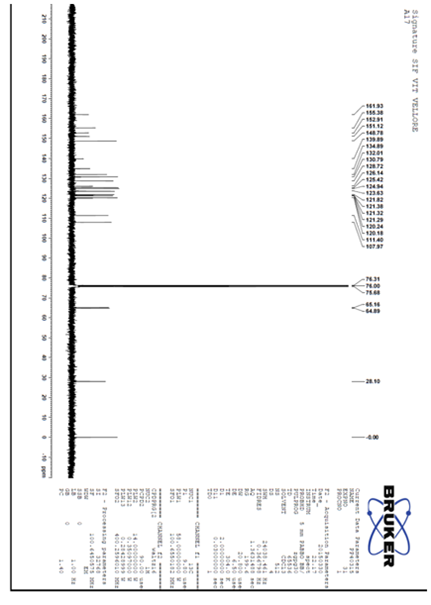

LC-MS of compound **6g**

IR of compound **6g**

^1^H NMR of compound **6h**


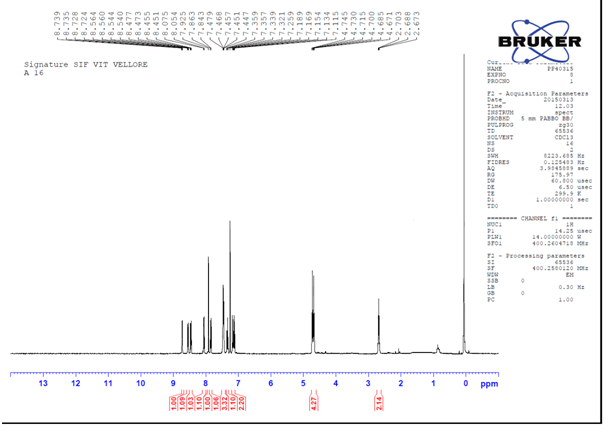

^13^C NMR of compound **6h**


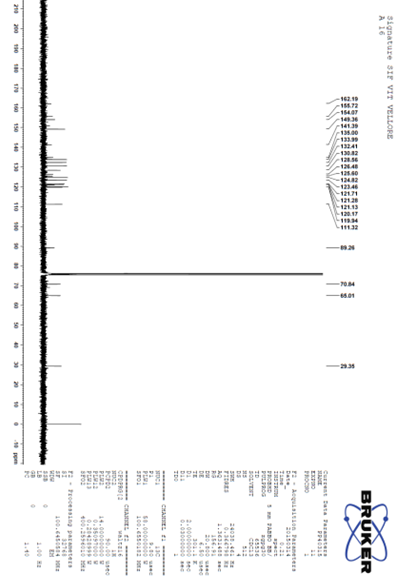

LC-MS of compound **6h**

IR of compound **6h**

^1^H NMR of compound **6i**


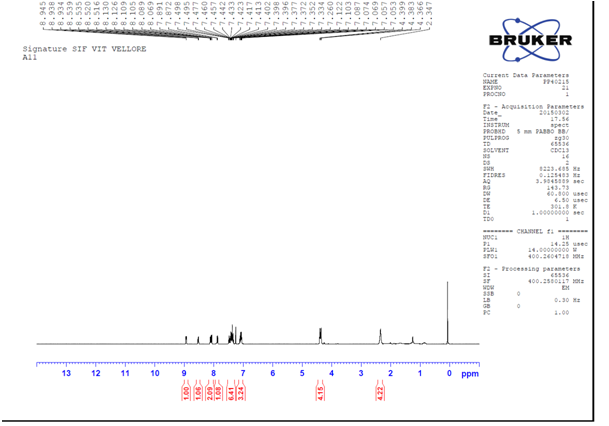

^13^C NMR of compound **6i**


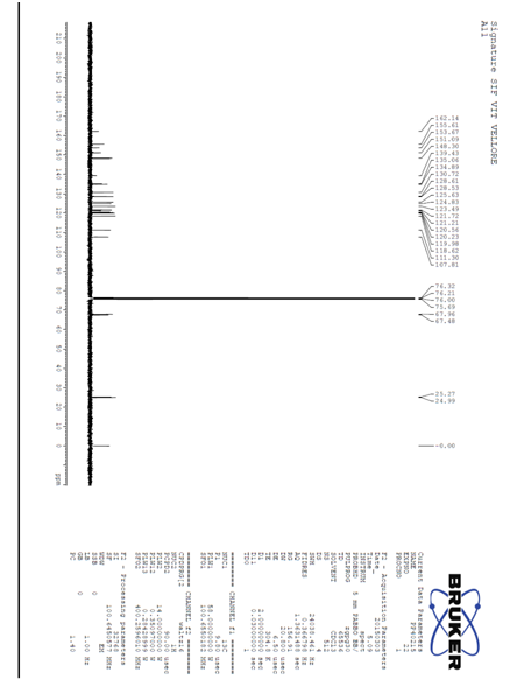

LC-MS of compound **6i**

IR of compound **6i**

^1^H NMR of compound **6j**


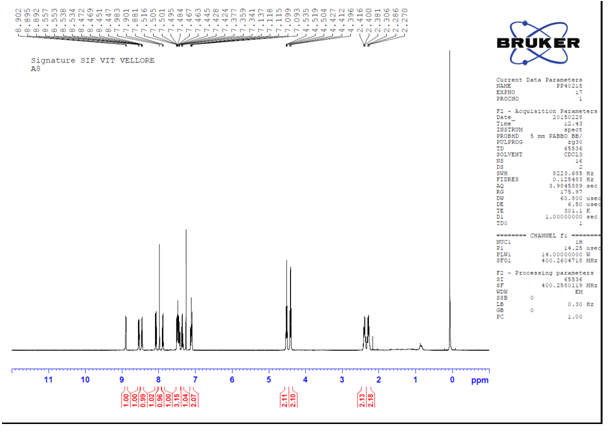

^13^C NMR of compound **6j**


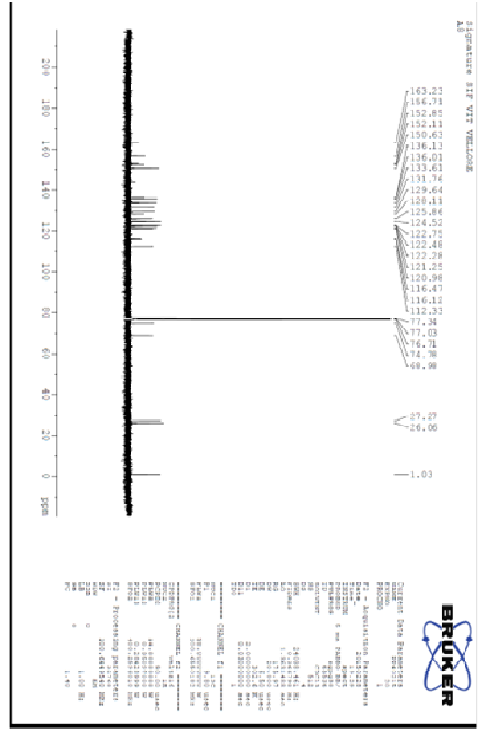

LC-MS of compound **6j**

IR of compound **6j**

^1^H NMR of compound **6k**


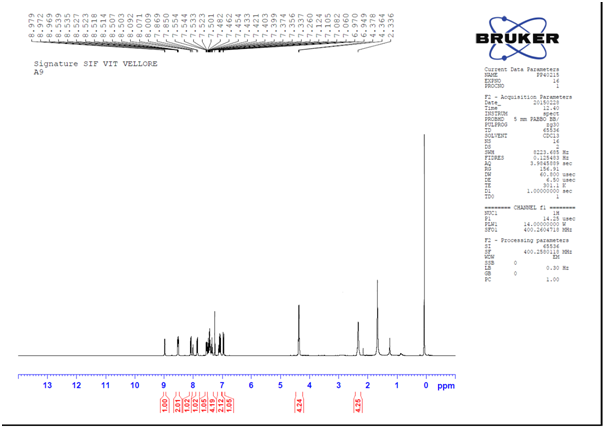

^13^C NMR of compound **6k**


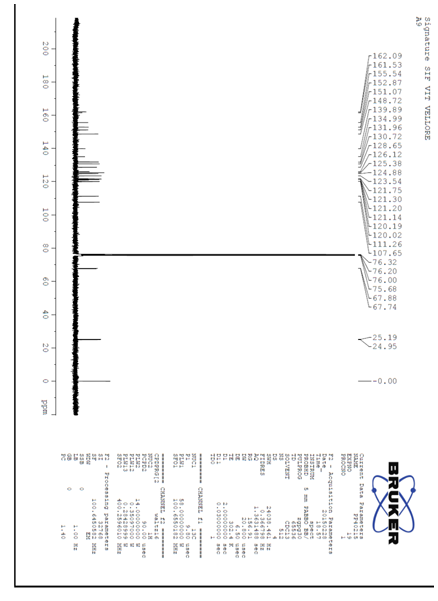

LC-MS of compound **6k**

IR of compound **6k**

^1^H NMR of compound **6l**


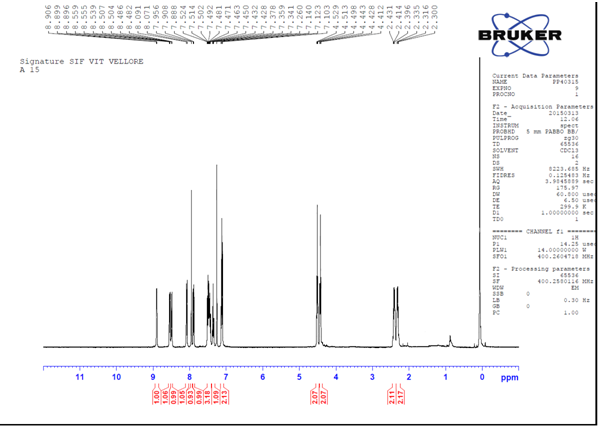

^13^C NMR of compound 6l


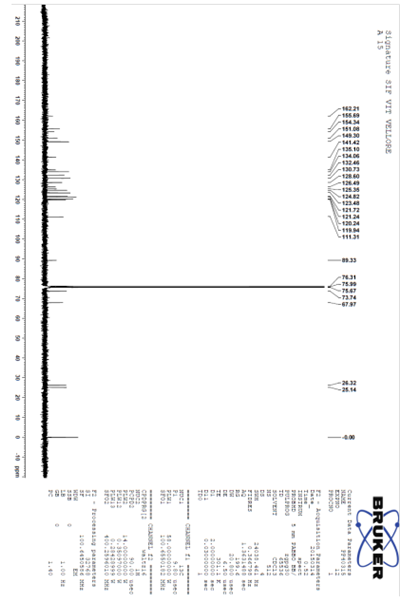

LC-MS of compound **6l**

IR of compound **6l**

**X-Ray Crystallographic Data**

Formula: C_24_ H_17_ Cl_1_ N_2_ O_2_ S_1_

Unit Cell Parameters: a 11.4545(11) b 11.5145(11) c 12.0254(12) P-1

******

**
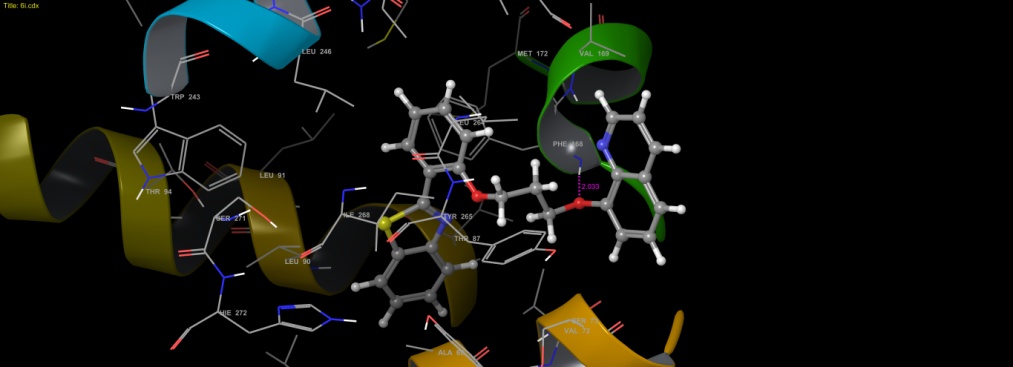

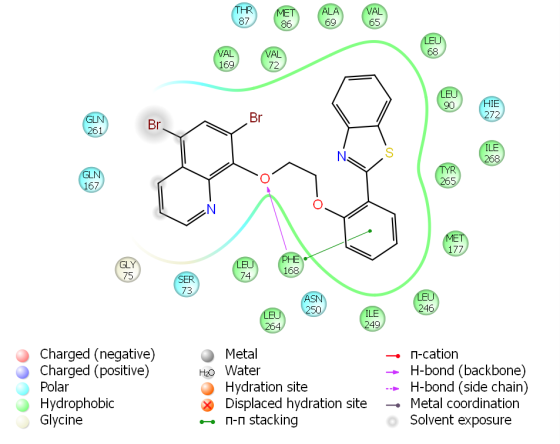
**

**(a)**

**
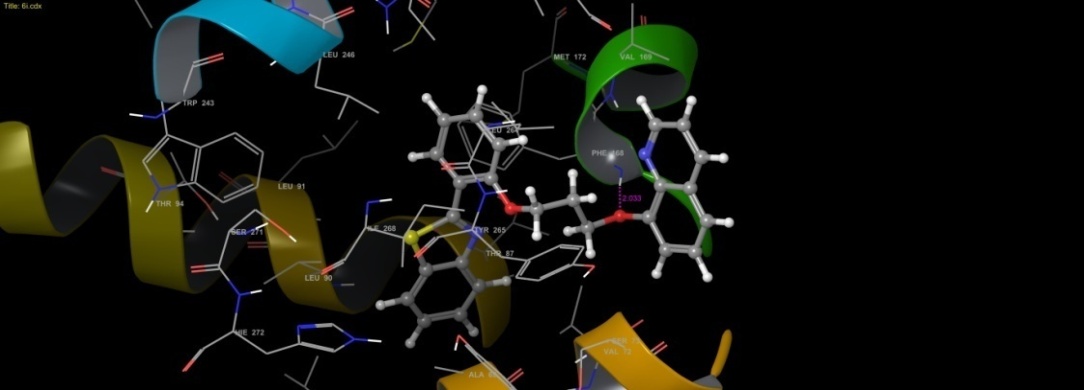

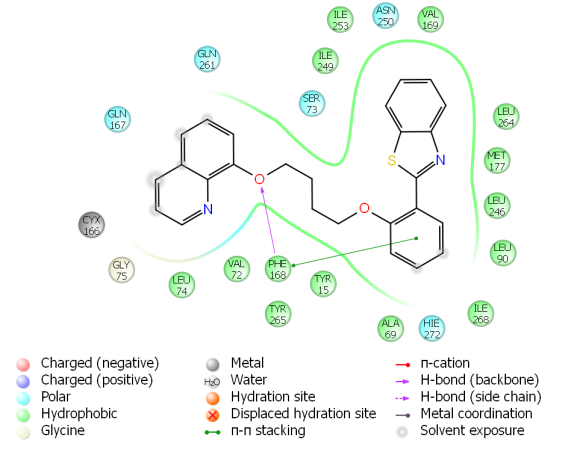
**

**(b)**

**
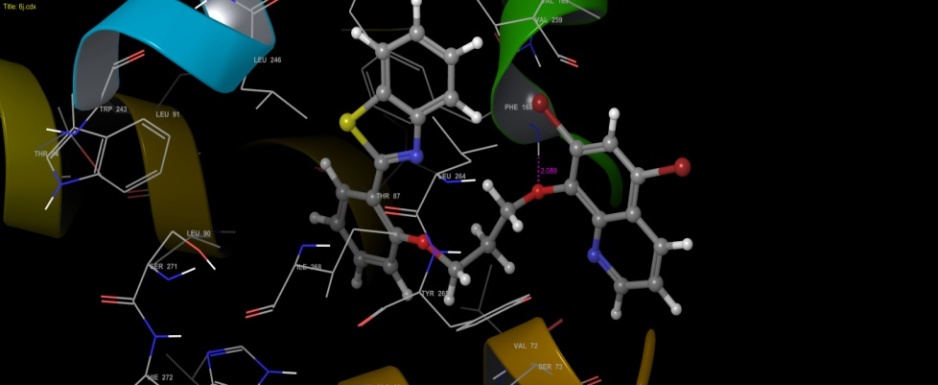

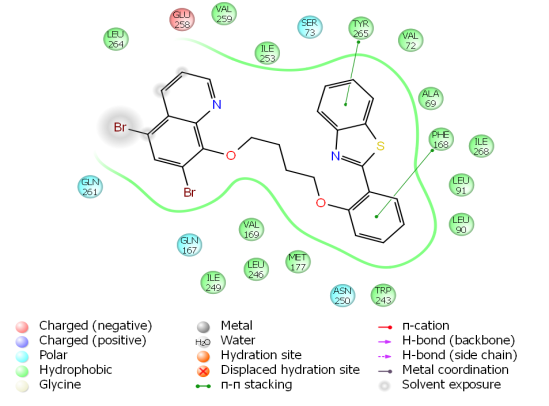
**

**(c)**

**
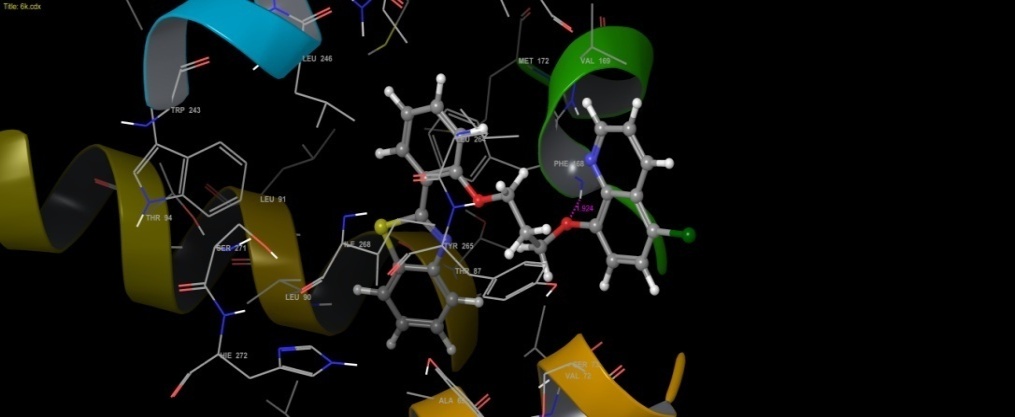

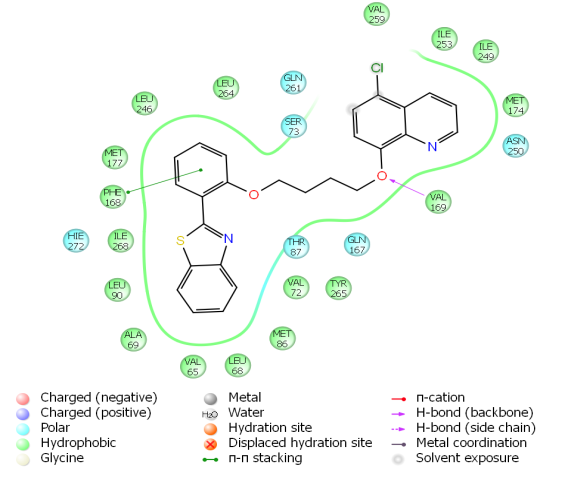
**

**(d)**

**
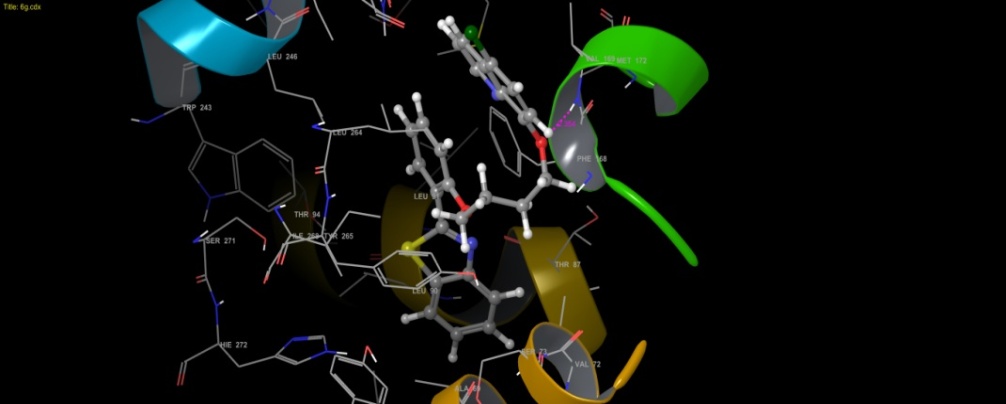

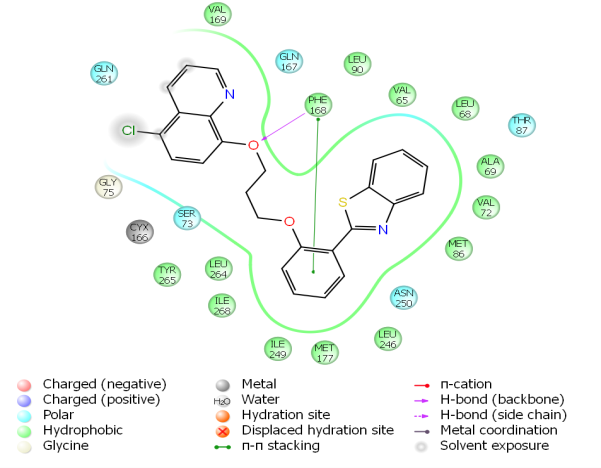
**

**(e)**

**
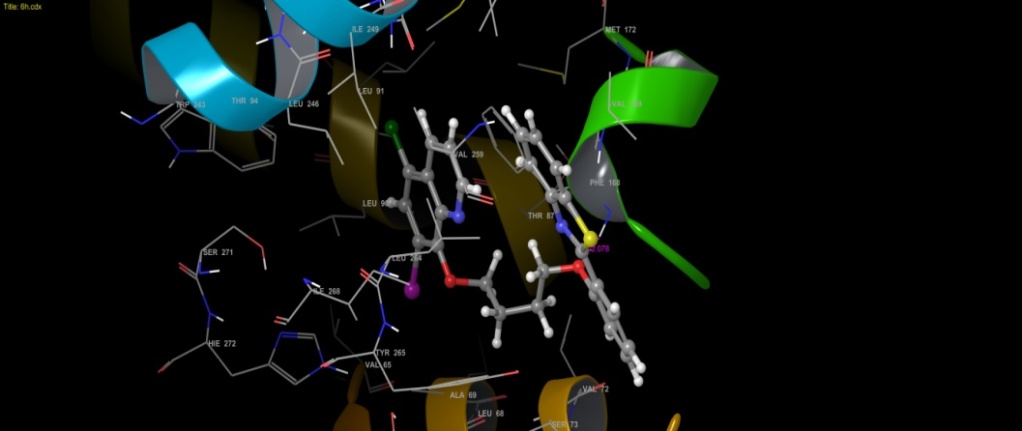

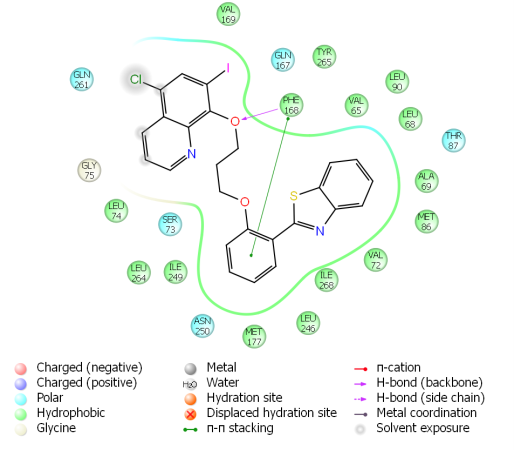
**

**(f)**

**Figure S1.** Binding interactions of selected benzothiazolylquinoline analogues (a) **6b** (b) **6e** (c) **6f** (d) **6g** (e) **6k** (f) **6l** with *h*A_3_ receptor.
